# In search of prosociality in rodents: A scoping review

**DOI:** 10.1371/journal.pone.0310771

**Published:** 2024-11-07

**Authors:** Valérie Charron, Joey Talbot, Patrick R. Labelle, Anne T. M. Konkle, Hélène Plamondon

**Affiliations:** 1 Behavioural Neuroscience Group, School of Psychology, University of Ottawa, Ottawa, Ontario, Canada; 2 Interdisciplinary School of Health Sciences, University of Ottawa, Ottawa, Ontario, Canada; 3 University of Ottawa Library, University of Ottawa, Ottawa, Ontario, Canada; 4 University of Ottawa Brain and Mind Research Institute, Ottawa, Ontario, Canada; Universidade do Porto Instituto de Biologia Molecular e Celular, PORTUGAL

## Abstract

Studying prosociality in rodents can provide insight into brain mechanisms potentially related to neurodevelopmental disorders known to impact social behaviors (e.g., autism spectrum disorder). While many studies have been published suggesting promising models, current knowledge remains scattered, including potential factors mediating prosocial behaviors in rodents. Prosocial behavior is characterized by an action done to benefit another or promote their well-being. The goal of this scoping review is to characterize current findings regarding prosocial paradigms in rodents, highlight current gaps in reporting, and identify factors shown to be important in mediating prosocial responses in rodents. Five databases were consulted in search of relevant studies published between 2000 and 2020 (APA PsycInfo, Embase, MEDLINE, Scopus, Web of Science). An update using a semi-supervised machine learning approach (ASReview) was then conducted to collect studies from 2021–2023. In total, 80 articles were included. Findings were the following: (1) Three categories of prosocial paradigm were extracted: cooperation, helping, and sharing tasks, (2) Rodents showed the ability to perform prosocial actions in all three categories, (3) Significant gaps in reported methodologies (e.g., failure to report animals’ characteristics, housing conditions, and/or experimental protocol) and mediating factors (e.g., sex, strain, housing, food restriction) were found, and (4) Behaviors are determinant when investigating prosociality in rodents, however many studies omitted to include such analyses. Together these results inform future studies on the impact of mediating factors and the importance of behavioral analyses on the expression of prosocial behaviors in rodents.

## Introduction

While there has been extensive research in humans [1], investigating prosociality in animals can present many challenges as their internal state can only be assumed and interpreted [2]. Rodents, prominently rats and mice are often used because of their sociability (e.g., social interaction seeking, hierarchical structure) [3] and practicality (reduced costs and resources compared to non-human primates) [4]. Aversive conditions such as pain or fear have been interpreted in rats and mice through operationalized nonverbal cues (e.g., freezing behaviors) and related tasks (e.g., emotional contagion). However, there has not yet been a standardized way to measure positive affect through behavioral indicators [2]. As such, prosocial tasks can be useful since they require an explicit action (e.g., pressing a lever, nose poking). Prosocial behavior is characterized by an action done to benefit another or promote their well-being [2]. Acting in a way that is prosocial is considered important to the survival of many species, such as a social group cooperating for resources [2, 5, 6]. Understanding prosociality in animals is crucial to gain deeper insights on endogenous mechanisms related to neuropsychiatric disorders that are characterized by social impairments (e.g., autism spectrum disorder, schizophrenia, personality disorders) [4, 7, 8].

### Rodent prosocial paradigms

Research examining prosociality in rodents began with the use of observational tasks, such as fear conditioning and emotional contagion paradigms [9–11]. These aversive models generally involve two animals, one receiving a painful stimulus (e.g., electric foot shock, tail pinch) while the other observes [12–14]. While these paradigms can be useful to study observational learning and the ability to respond to the distress of another conspecific, these models primarily focus on examining survival responses (i.e., learning from the environment and others to avoid danger/an aversive situation) while offering limited insights into prosocial responses [14, 15]. It is an important distinction to be made as the primary incentive driving these behaviors differ, the former being a survival response while the latter involves a higher level of decision-making [16]. In 2011, Ben-Ami Bartal and colleagues published a groundbreaking study in which rats learned to open a door to free a conspecific from a restraining device [17]. Since then, minimally aversive paradigms have been implemented, including other variations of freeing task (e.g., freeing a conspecific from a pool of water), and a prosocial choice task, in which a rat can choose between eating a reward on its own (own-reward compartment) or eating it with a conspecific, who also receives a reward (both-reward compartment) [18, 19]. The development of non-aversive paradigms becomes imperative as welfare is gaining importance in animal studies [20]. Moreover, many highlighted the possibility that the absence of an aversive variable (i.e., foot shock), eliminating the ‘survival instinct’ factor, could better represent prosocial behaviors [16]. To date, objective assessments of non-aversive models have been challenging [21, 22], and some have raised questions as to whether recorded observations truly represent prosociality rather than the simple desire of a reward (i.e., social contact, food) [23, 24]. Attempts to address this issue have included preventing social contact between rodents using a wire mesh [25] or freeing a conspecific in a separate compartment [26]. Other studies have included quantitative metrics, such as discriminant analysis of behaviors [27] or recording of ultrasonic vocalizations [17] to help determine prosociality beyond task performance.

### Mediating factors in the expression of prosociality in rodent

Prosociality being a complex behavior, multiple mediating factors have been suggested as potentially impacting rodents’ expression of prosocial behaviors [27, 28]. Such factors include individual characteristics (i.e., sex, age, strain) [27–29], dominance and social hierarchy [30, 31], familiarity [17, 31], characteristics of the paradigm (i.e., reward, task contingency, social contact) [32, 33], housing conditions [34], and stress (i.e., handling and experimental procedures such as surgeries) [35, 36]. For example, rodents that are housed in pairs tend to develop a role of dominance or submission, which tend to be determined by various factors, including the sex and weight of the animal [37]. Furthermore, stress levels have a direct impact on dyadic relationships, with the stressed rat being more submissive [38]. Moreover, rodents tend to display more prosocial behaviors with other familiar animals versus strangers [12], and with same-strain congeners [17]. Interestingly, adolescent rats tend to be prosocial with ingroup and outgroup members, whereas adult rats tend to display prosociality with ingroup members only [29]. These examples show that various mediating factors can play a significant role in the expression of prosocial behaviors in rodents.

### Research objectives

Although interesting studies have been published suggesting promising paradigms [7, 17, 19], current knowledge remains scattered, including potential factors mediating prosocial behaviors in rodents. Therefore, the goal of this scoping review is to characterize current findings regarding prosocial paradigms in rodents, highlight current gaps in reporting, and identify factors shown to be important in mediating prosocial responses in rodents. Furthermore, the review sought to characterize and define categories of paradigms often used in the field of prosocial research (cooperation, helping, sharing). In this review, the focus is placed on prosocial tests, which are defined as tasks that include a concrete action toward a congener for individual or mutual benefit. As such, the following search strategy is based solely on articles using a task requiring two or more rodents and a specific action.

## Methodology

The methodology of this review was registered in PROSPERO (registration number: CRD42022335883). We followed the Preferred Reporting Items for Systematic reviews and Meta-Analyses guidelines (PRISMA) protocol and flowchart. Covidence (Veritas Health Innovation) was used to manage references.

### Search strategy

The original search strategy sought to identify studies on prosociality in rodents. A research librarian (PRL) with experience in planning reviews drafted, developed, and implemented a search strategy to find pertinent published articles in APA PsycInfo (Ovid), Embase (Ovid), MEDLINE (Ovid), Scopus and Web of Science (Clarivate). The strategy was informed by ones conducted in previous reviews on rodents, mice and rats [39–41] and on prosociality [42–45]. A draft strategy, which included subject headings and keywords, was developed for APA PsycInfo (Ovid) by the research librarian and feedback was obtained from other review team members. The strategy was also peer-reviewed by another librarian following the Peer-Review of Electronic Search Strategy guideline [46]. The initial searches were executed on January 26, 2021. The search did not use any database limits other than a date restriction, limiting results to those published since 2000. The complete search strategy is available in S1 Appendix.

**Fig 1:**
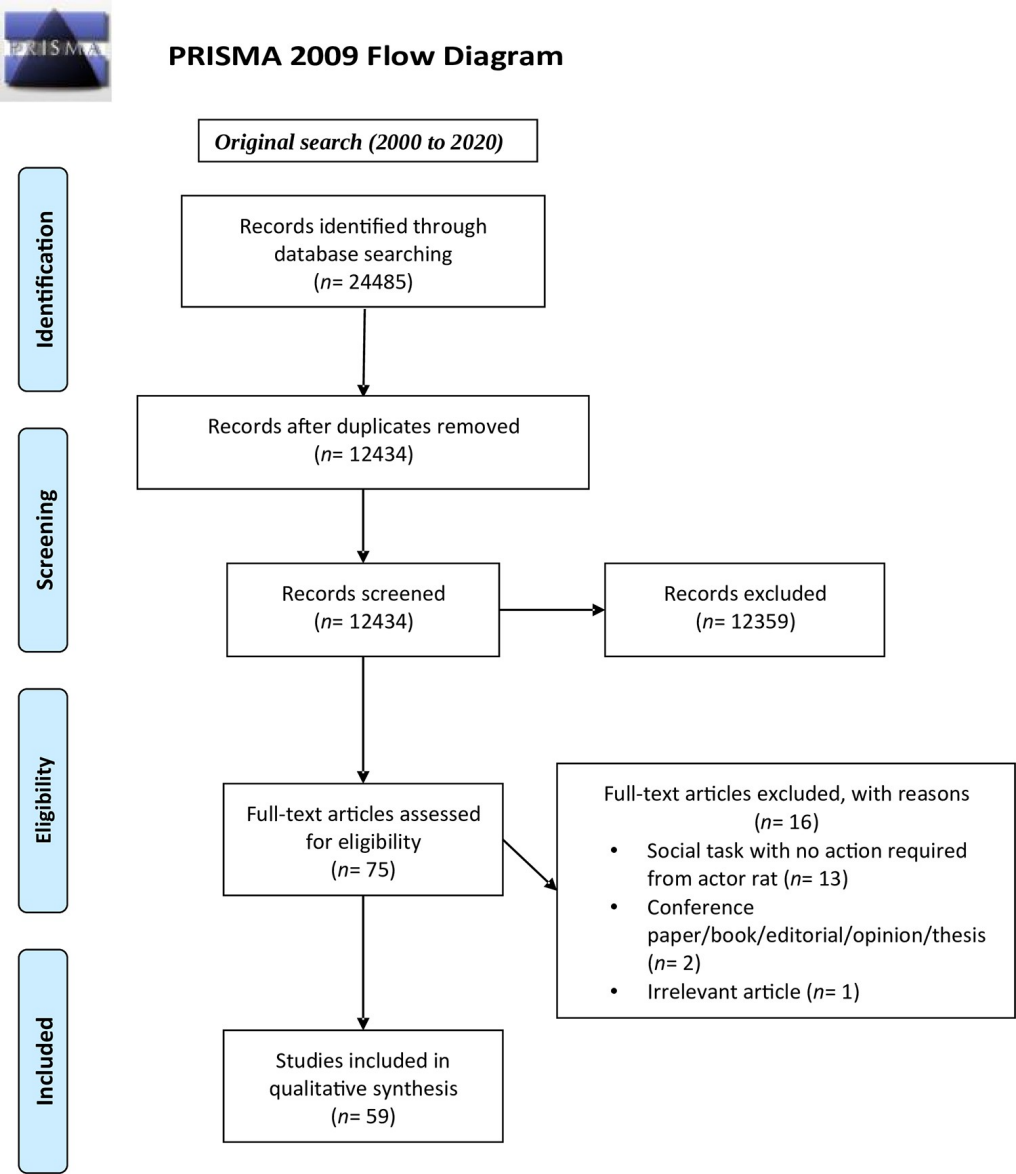
PRISMA flow diagram.

Citations found through the database searches were imported into Covidence, an online tool used to manage various steps of a review’s screening phases. A total of 24485 published articles between January 1st, 2000, and December 31^st^, 2020, were found. Duplicate references were identified and removed once imported into Covidence. Additional duplicates were identified and excluded while screening references. After duplicate removal, 12434 articles were left for abstract screening (Fig 1). All inclusion and exclusion criteria can be found in Table 1 and all information to be extracted from each study can be found in Table 2.

**Table 1:** Inclusion and exclusion criteria for the scoping review.

Inclusion criteria	Exclusion criteria
Rats	Animals other than rodents (mole rats, guinea pigs, primates, humans, etc.)
Mice	Study not published in English
Study published in English	Study published before 2000
Study published between 2000–2020	Conference paper, thesis, opinion/editorial paper or books
The task includes two or more animals (including inanimate control or conditions)	Scoping review, systematic review or meta-analysis
The task includes an action for at least one rat (no passive tasks)	Social task that requires no action from rats (social interaction test, open field, novelty tasks–no prosocial components)
	Pregnancy or maternal/paternal studies
	Study Erratum
	Irrelevant studies
	Study not found

**Table 2:** Details to be extracted from each included study.

Category	Extracted data	Details
Article journal	● Title ● Authors names ● Date of publication ● Journal ● Open access ● Definition of the task	Open access: Is the article published in an open access journal? yes or no Definition of the task: Report any definitions that authors have used to refer to prosociality or the task
Animals	● Ethical note ● Type of rodent ● Total # of subjects ● Total of males/females ● Age at arrival ● Strain ● Weight at arrival ● Food restriction ● Water restriction	Ethical note: Including the name of the animal care committee, the license number if applicable and the followed guidelines Type of rodent: Rats or mice Total of males/females: In case only one sex was tested, ‘NA’ is indicated for the non relevant sex Food and water restriction: Yes or no,—if yes, precision is given (e.g., 85% of free feeding body weight)
Housing conditions	● Number of animals per cage ● Relationship ● Dark-light cycle ● Enriched environment	Number of animals per cage: Modalities of animal housing–ex: Were the animals housed in pairs or more? If housed with more than one individual, specify whether the animal is housed with its experimental partner Relationship: Are the animals’ littermates or unrelated? Dark-light cycle: Indicate if the investigators specified the light/dark cycle (e.g., 12h, 16h, reversed) Enriched environment: Did the investigators use an enriched environment (yes or no). If yes, details if available are specified (description of the environment)
Methodology	● Operant paradigm ● Total of rats involved ● Groups ● Description of the task ● Type of task ● Computerized task ● Aversive or non-aversive ● Sensory paradigm (can rodents see/hear/smell each other) ● Duration of task (in minutes) ● Total of testing days ● Pretraining/habituation ● Control Group	Operant paradigm: Precise the selected operant paradigm (e.g., prosocial choice task, prisoner’s dilemma, freeing task, repeated donation game, etc.) Groups: Precise the group repartition Description of the task: Summary of the selected task Type of task: Cooperation, sharing, freeing, etc. Computerized task: Does the paradigm use an automated or computerized system? (e.g., operant box, video recording, ultrasonic vocalization, etc.) Aversive or non-aversive: Does the paradigm involve an aversive component (e.g., pain, fear, distress); if so, for one or both rats? (yes or no) Sensory elements: Do authors mention if rodents can see, hear or smell each other during the experimental task? (yes or no) Pretraining/Habituation: Is there a mention of a habituation or pretraining phase? (if yes, describe) Control group: Is a control group included? (if yes, describe)
Experimental interventions	● Drugs ● Surgery ● Diet ● Stress ● Other	Experimental interventions: Describe any use of drugs, diet, stress variables, surgery or other tests done during the course of the experiment (e.g., open field, elevated plus maze, etc.)
Results	● Performance index ● Behavioral analyses ● Sex differences ● Age differences ● Strain differences	Performance index: How did the study quantify the action? (e.g., lever presses, door openings, etc.) Behavioral analyses: Did the study use any metrics to analyze rodents’ behaviors? (e.g., video recording, ultrasonic vocalization) if yes, summarize the results. Sex differences: If the study used both sexes, summarize sex differences’ results if applicable Age differences: Summarize any results regarding age differences if applicable Strain differences: Summarize any results regarding strain differences if applicable

### Update using ASReview

**Fig 2:**
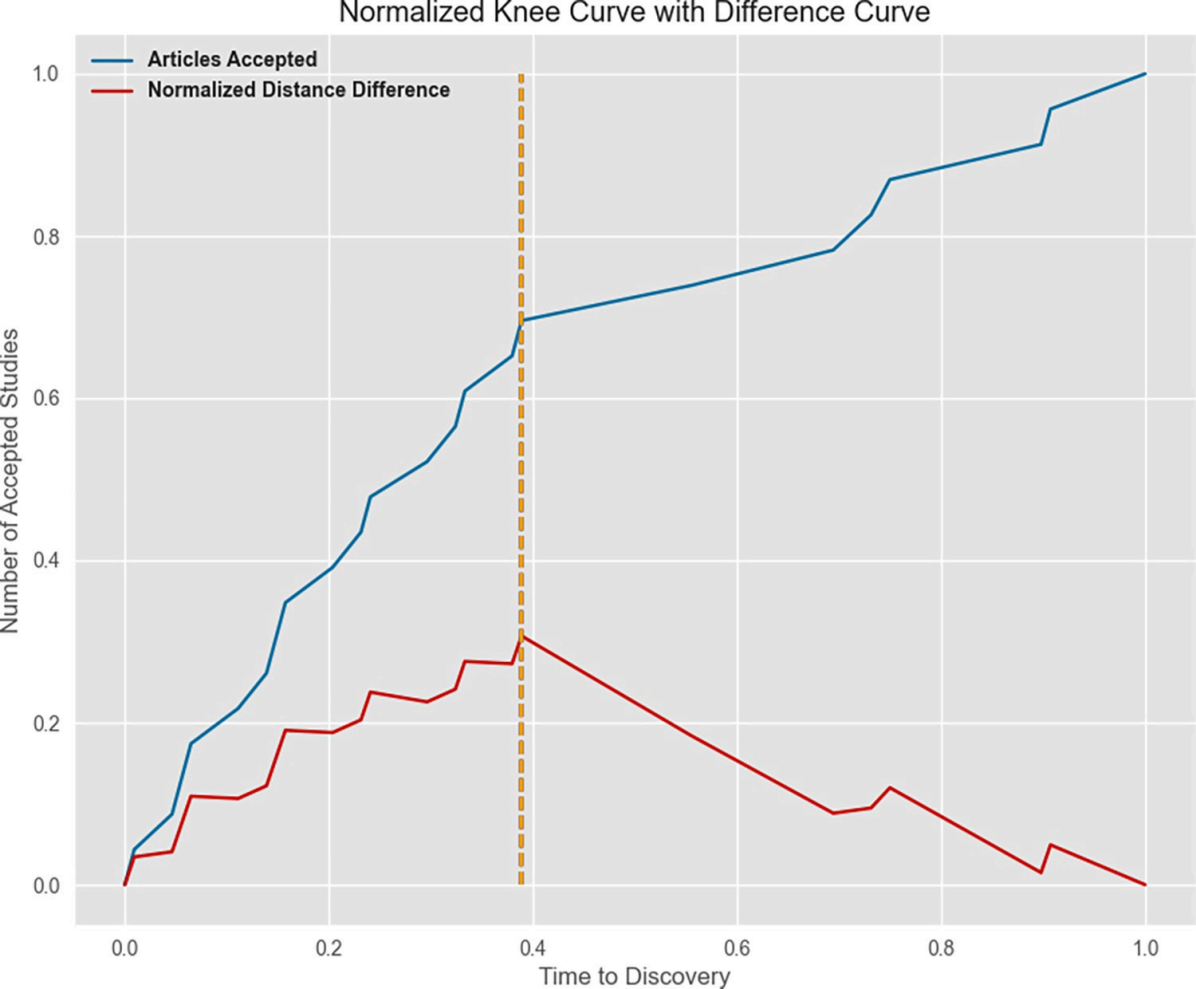
Stopping rule: Knee curve. The knee is located at the 17th accepted article (time of discovery: 43 articles) which is represented by the dotted line. In total, 128 articles were screened (represented by the blue line) and 21 were accepted for data extraction.

An update from January 1, 2021, to January 17th, 2023, was performed by means of a semi-supervised machine learning approach. The software used for this purpose, ASReview [47] calculates the relevancy of studies based on a trained semi-supervised machine learning algorithm. The same search strategy and search phrases were used to establish the updated database. A total of 4071 articles were found. After duplicate removal, 2001 articles were left for abstract screening (see Fig 2). Using the open-source ASReview software, the semi-supervised machine learning model was trained (by either accepting or rejecting studies) by the two principal authors using the original database of 12432 articles. The original dataset (the training data) was then imported into the ASReview software for the review update. The ASRreview software presents relevant articles to the researchers early in the screening process, and depending on the stopping rule, allows the researchers to end the screening process earlier than other methods such as manual screening [47]. The use of such methods allows for faster screening and higher accuracy in the identification of relevant studies [47]. When conducting a scoping review with ASReview, settings need to be chosen by the researcher based on the needs and goals of the study: 1) The algorithm for feature extraction, 2) classifying algorithm, 3) balancing strategy, 4) Querying strategy. Chosen settings were the following: 1) the SentenceBert algorithm in conjunction with 2) a logistic regression for its classifier [48, 49], 3) a double dynamic resampling strategy to prevent model overfitting, and 4) a mixed querying strategy consisting of a 95% maxed matching and a 5% random study presentation.

**Fig 3:**
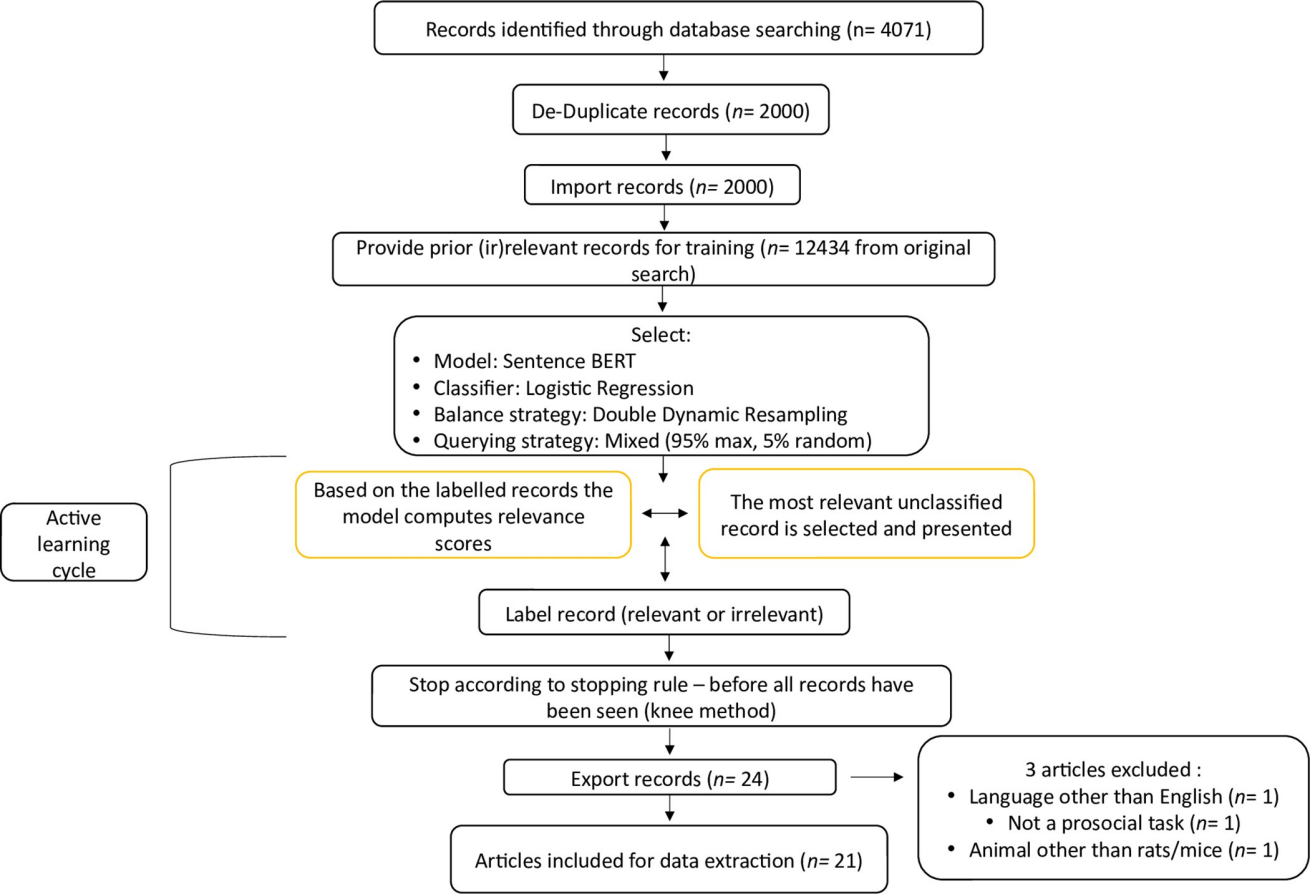
ASReview flow diagram.

A knee detection algorithm was actively applied throughout the screening process to identify an appropriate stopping point. Within the screening process, a stopping point is when most of the potentially relevant studies have been identified and accepted, and the probability of unscreened studies to contain a relevant study is low [50]. A sensitivity of 5 was selected, (the default being a value of 1), to be more conservative in the stopping time, thus increasing the probability of accepting relevant studies [50]. The increasing concave curve option was chosen to visually represent the rising number of screened studies. For detailed explanation of the Kneedle algorithm and parameters, see the original paper [50]. Once the knee was detected by the Kneedle algorithm, screening continued for a short time to confirm that the initial knee was correct [50]. The location of the knee was at the 17th accepted article (time of discovery: 43 articles; Fig 2). In total, 128 articles were screened, representing 6.39% of the 2001 articles. Amongst these, 21 articles were included for data extraction (Fig 3).

### Risk of bias tool

This review used an adapted version of SYRCLE risk of bias tool for animal studies to assess the methodological quality of included studies [51]. Two independent reviewers (two principal authors) assessed the articles, and any disagreement was resolved through consensus-oriented discussion or by consulting a third party (principal investigator) [51]. SYRCLE is a tool designed for randomized control trial (RCT) studies. Therefore, different aspects pertaining to RCT designs were not evaluated in this review, considering that many of the included studies could be defined as observational studies rather than RCTs.

### Statistical analyses

Descriptive statistics (e.g., averages, percentages) of the extracted data were performed to identify key information (e.g., number of rats, sex, strain). When studies used similar paradigms (i.e., freeing task, food sharing task), descriptive statistics were used to compare and discuss them. Key components of every paradigm were extracted and described using qualitative comparisons (e.g., task, rewards, operant chamber, etc).

## Results

The 80 included articles (*n* = 59 from original search) were organized into 3 categories (i.e., cooperation, helping, or sharing task) and classified as either using an aversive (*n* = 38) or a non-aversive (*n* = 42) testing paradigm (see Table 3). An aversive task is characterized by inclusion of an aversive element causing distress or discomfort to the animals, such as a restraining device, water exposure, electric shocks or tail pinches. All details regarding the data extraction for each included article can be found in supplementary data (S2 Appendix).

**Table 3:** Included articles.

Study	Type of rodent	Type of task	Aversive or non-aversive
**Original search (2000 to 2020)**			
Avital, Aga-Mizrachi & Zubedat, 2016 [52]	Rats	Cooperation (learning task)	Non aversive
Bartal, Decety & Mason, 2011 [17]	Rats	Helping (freeing task–tube)	Aversive (restraint)
Bartal, Rodgers, Sarria, Decety, & Mason, 2014 [53]	Rats	Helping (freeing task–tube)	Aversive (restraint)
Bartal, Shan, Molasky, Murray, Williams, Decety & Mason, 2016 [54]	Rats	Helping (freeing task–tube)	Aversive (restraint)
Blystad, Andersen & Johansen, 2019 [32]	Rats	Helping (freeing task–tube)	Aversive (restraint)
Carvalheiro, Seara-Cardoso, Mesquita, de Sousa, Oliveira, Summavielle & Magalhães, 2019 [55]	Rats	Helping (freeing task–tube)	Aversive (restraint)
Conde-Moro, Rocha-Almeida, Sánchez-Campusano, Delgado-García & Gruart & 2019 [56]	Rats	Cooperation (learning task)	Non aversive
Cox & Reichel, 2020 [26]	Rats	Helping (freeing task–soaked area)	Aversive (water)
Daghestani, Selim, Abd-Elhakim, Said, Abd-Hameed, Khalil & El-Tawil, 2017 [57]	Rats	Helping (freeing task–tube)	Aversive (restraint)
de Carvalho, Dos Santos, Regaço, Barbosa, Da Silva, de Souza & Sandaker, 2018 [58]	Rats	Cooperation (learning task)	Non aversive
Delmas, Lew & Zanutto, 2019 [59]	Rats	Cooperation (Prisoner’s dilemma)	Non aversive
Dolivo & Taborsky 2015 (A) [60]	Rats	Sharing (repeated donation game)	Non aversive
Dolivo & Taborsky 2015 (B) [61]	Rats	Sharing (repeated donation game)	Non aversive
Donovan, Ryan & Wood, 2020 [62]	Rats	Cooperation (Prisoner’s dilemma)	Non aversive
Festucci, Buccheri, Cerniglia, Paciello, Cimino, Curcio & Adriani, 2020 [63]	Rats	Sharing task (repeated donation game)	Non aversive
Fontes-Dutra, Nunes, Santos-Terra, Souza-Nunes, Bauer-Negrini, Hirsch, Green, Riesgo, Gottfried & Bambini-Junio, 2019 [64]	Rats	Helping (freeing task–tube)	Aversive (restraint)
Gerber, Schweinfurth & Taborsky, 2020 [65]	Rats	Sharing task (repeated donation game)	Non aversive
Hachiga, Schwartz, Silberberg, Kearns, Gomez & Slotnick, 2018 [66]	Rats	Helping (freeing task–tube)	Aversive (restraint)
Han, Yoon, Shin, Um & Ko, 2020 [67]	Mice	Cooperation (learning task)	Non aversive
Havlik, Sugano, Jacobi, Kukreja, Jacobi & Mason, 2020 [68]	Rats	Helping (freeing task–tube)	Aversive (restraint)
Hernandez-Lallement, van Wingerden, Marx, Srejic & Kalenscher, 2015 [19]	Rats	Sharing (prosocial choice task)	Non aversive
Hernandez-Lallement, van Wingerden, Schäble & Kalenscher, 2016 [69]	Rats	Sharing (prosocial choice task)	Non aversive
Hernandez-Lallement, Attah, Soyman, Pinhal, Gazzola & Keysers, 2020 [70]	Rats	Helping (not harming)	Aversive (electric shocks)
Hosgorler, Koc, Kizildag, Canpolat, Argon, Karakilic, Kandis, Guvendi, Ates, Arda & Uysal, 2020 [71]	Rats	Helping (freeing task–soaked area)	Aversive (water)
Kandis, Ates, Kizildag, Camsari, Yuce, Guvendi, Koc, Karakilic, Camsari & Uysal, 2018 [72]	Rats	Helping (freeing task–soaked area)	Aversive (water)
Karakilic, Kizildag, Kandis, Guvendi, Koc, B. Camsari, M. Camsari, Ates, Arda & Uysal, 2018 [6]	Rats	Helping (freeing task–soaked area)	Aversive (water)
Kentrop, Kalamari, Danesi, Kentrop, van IJzendoorn, Bakermans-Kranenburg, Joëls & van der Veen, 2020 [73]	Rats	Sharing (lever presses)	Non aversive
Kozma, Kassai, Ernyey & Gyerytán, 2019 [74]	Rats	Cooperation (learning task)	Non aversive
Li & Wood, 2017 [75]	Rats	Sharing (repeated donation game)	Non aversive
Lopuch & Popik, 2011 [25]	Rats	Cooperation (learning task)	Non aversive
Márquez, Rennie, Costa & Moita, 2015 [76]	Rats	Sharing (prosocial choice task)	Non aversive
Oberliessen, Hernandez-Lallement, Schäble, van Wingerden, Seinstra & Kalenscher, 2016 [77]	Rats	Sharing (prosocial choice task)	Non aversive
Raz, 2013 [78]	Rats	Cooperation (learning task)	Non aversive
Rutte & Taborsky, 2007 [79]	Rats	Sharing (lever presses)	Non aversive
Rutte & Taborsky, 2008 [80]	Rats	Sharing (repeated donation game)	Non aversive
Sato, Tan, Tate & Okada, 2015 [18]	Rats	Helping (freeing task–soaked area)	Aversive (water)
Schmid, Schneeberger & Taborsky, 2017 [81]	Rats	Sharing (stick pulling)	Non aversive
Schneeberger, Dietz & Taborsky, 2012 [82]	Rats	Sharing (repeated donation game)	Non aversive
Schönfeld, Schäble, Zech & Kalenscher, 2020 [83]	Rats	Sharing (prosocial choice task)	Non aversive
Schwartz, Silberberg, Casey, Kearns & Slotnick, 2017 [84]	Rats	Helping (freeing task—soaked area)	Aversive (water)
Schweinfurth & Taborsky, 2016 [85]	Rats	Sharing (repeated donation game)	Non aversive
Schweinfurth & Taborsky, 2017 [86]	Rats	Sharing (repeated donation game)	Non aversive
Schweinfurth & Taborsky, 2018 (A) [87]	Rats	Sharing (repeated donation game)	Non aversive
Schweinfurth & Taborsky, 2018 (B) [88]	Rats	Sharing (repeated donation game)	Non aversive
Schweinfurth & Taborsky, 2018 (C) [89]	Rats	Sharing (stick pulling)	Non aversive
Schweinfurth, Aeschbacher, Santi & Taborsky, 2019 [90]	Rats	Sharing (repeated donation game)	Non aversive
Schweinfurth & Taborsky, 2020 [91]	Rats	Cooperation (Prisoner’s dilemma)	Non aversive
Silberberg, Allouch, Sandfort, Kearns, Karpel & Slotnick, 2014 [24]	Rats	Helping (freeing task–soaked area)	Aversive (water)
Silva, H. Silva, Lima, Meurer, Ceppi & Yamamoto, 2020 [92]	Rats	Helping (freeing task–tube)	Aversive (restraint)
Tomek, Stegmann & Olive, 2019 [93]	Rats	Helping (freeing task–tube)	Aversive (restraint)
Tomek, Stegmann, Leyrer-Jackson, Piña & Olive, 2020 [94]	Rats	Helping (freeing task–tube)	Aversive (restraint)
Tsoory, Youdim & Schuster, 2012 [95]	Rats	Cooperation (learning task)	Non aversive
Ueno, Suemitsu, Murakami, Kitamura, Wani, Matsumoto, Okamoto & Ishihara, 2019 (A) [96]	Mice	Helping (freeing task–tube)	Aversive (restraint)
Ueno, Suemitsu, Murakami, Kitamura, Wani, Matsumoto, Okamoto & Ishihara, 2019 (B) [97]	Mice	Helping (freeing task–tube)	Aversive (restraint)
Viana, Gordo, Sucena & Moita, 2010 [98]	Rats	Cooperation (Prisoner’s dilemma)	Aversive (tail pinches)
Wood, Kim & Li, 2016 [99]	Rats	Cooperation (Prisoner’s dilemma)	Non aversive
Yamagishi, Okada, Masuda & Sato, 2020 (A) [100]	Rats	Helping (freeing task–soaked area)	Aversive (water)
Yamagishi, Lee & Sato, 2020 (B) [101]	Rats	Helping (freeing task–soaked area)	Aversive (water)
Yüksel, Ates, Kizildag, Yüce, Koç, Kandis, Güvendi, Karakilic, Gümüs & Uysal, 2019 [102]	Mice	Helping (freeing task–soaked area)	Aversive (water)
**Updated search (2021 to 2023)**	**Type of rodent**	**Type of task**	**Aversive or non-aversive**
Asadi, Khodagholi, Asadi, Kamsorkh, Kaveh & Maleki, 2021 [103]	Rats	Helping (freeing task—soaked area)	Aversive (water)
Bartal, Breton, Sheng, Long, Chen, Halliday, Kenney, Wheeler, Frankland, Shilyansky, Deisseroth, Keltner & Kaufer, 2021 [104]	Rats	Helping (freeing task—tube)	Aversive (restraint)
Breton, Eisner, Gandhi, Musick, Zhang, Long, Perloff, Hu, Pham, Lalchandani, Barraza, Kantor, Kaufer & Bartal, 2022 [29]	Rats	Helping (freeing task—tube)	Aversive (restraint)
Conde-Moro, Rocha-Almeida, Gebara, Delgado-Garcia, Sandi & Gruart, 2022 [105]	Rats	Cooperation (learning task)	Non-aversive
Cox, Kearns, Woods, Brown, Brown & Reichel, 2022 [106]	Rats	Helping (freeing task—soaked area)	Aversive (water)
Cox, Brown, Woods, Brown, Kearns & Reichel, 2022 [107]	Rats	Helping (freeing task—soaked area)	Aversive (water)
de Carvalho, dos Santos, Regaço, Couto, de Souza & Todorov, 2020 [108]	Rats	Cooperation (learning task)	Non-aversive
Gachomba, Esteve-Agraz, Caref, Maroto, Bortolozzo-Gleich, Laplagne & Márquez, 2022 [30]	Rats	Sharing (prosocial choice task)	Non-aversive
Heslin & Brown, 2021 [109]	Rats	Helping (freeing task—tube)	Aversive (restraint)
Joushi, Taherizadeh, Esmaeilpour & Sheibani, 2022 [110]	Rats	Sharing (prosocial choice task)	Non-aversive
Kalamari, Kentrop, Danesi, Graat, van Ijzendoorn, Bakermans-Kranenburg, Joëls & van der Veen, 2021 [111]	Rats	Helping (freeing task—tube)	Aversive (restraint)
Misiolek, Klimczak, Chrószcz, Szumiec, Bryksa, Przyborowicz, Parkitna & Harda, 2023 [33]	Mice	Sharing (prosocial choice task)	Non-aversive
Paulsson & Taborsky, 2021 [112]	Rats	Sharing (repeated donation game)	Non-aversive
Scheggia, La Greca, Maltese, Chiacchierini, Italia, Molent, Bernardi, Coccia, Carrano, Zianni, Gardoni, Di Luca & Papaleo, 2022 [31]	Mice	Sharing (nose poking)	Non-aversive
Schweinfurth, 2021 [113]	Rats	Sharing (repeated donation game)	Non-aversive
Segura, Clavijo & Bouzas, 2019 [126]	Rats	Cooperation (learning task)	Non-aversive
Sen, Kara, Koyu, Simsek, Kizildag & Uysal, 2021 [35]	Rats	Helping (freeing task—soaked area)	Aversive (water)
Shima, Kawabata-Iwakawa, Onishi, Jesmin & Yoshikawa, 2022 [114]	Mice	Helping (freeing task—soaked area)	Aversive (water)
Subhadeep, Srikumar, Shankaranarayana & Kutty, 2022 [115]	Rats	Helping (freeing task—tube)	Aversive (restraint)
Wan, Kirkman, Jensen & Hackenberg, 2021 [116]	Rats	Helping (freeing task—tube)	Aversive (restraint)
Wu, Cheng, Liang, Lee & Yen, 2023 [117]	Rats	Helping (freeing task—tube)	Aversive (restraint)

The first category combines all cooperation tasks, such as the Prisoner’s dilemma task and the cooperation learning tasks (*n* = 16). The cooperation tasks involved two rodents that were trained to give a mutual response (lever presses, running through a maze) in a certain time interval to get a mutual reward. Another variation of cooperation tasks involved the Prisoner’s dilemma, in which both rodents have to decide to either cooperate or defect by pressing on a lever within a certain time interval.

The second category combines all helping tasks, including freeing tasks and tasks that prevent harm to a conspecific (*n* = 37). Helping tasks involved a rodent trapped in a restrainer tube or a soaked area while another conspecific could press a lever to open the door and free the animal. Another variant involved a rodent receiving electric shock while a conspecific could opt to press a lever to stop the shocks or to get a reward for itself.

The third and final category combines all sharing tasks, such as the prosocial choice task and the repeated donation game (*n* = 27). The latter is characterized by two rodents that can press a lever or pull a stick to give each other food rewards, while the prosocial choice task gives to a rodent the possibility to eat a food reward in an *own-reward* compartment or in a *both-reward* compartment (providing a reward to itself and a conspecific).

### Bias assessment

Results from the bias assessment can be found in Table 4. More details on risk of bias by category of task can be found in the discussion.

**Table 4:** Risk of bias assessment—Results (*n* = 80 included articles).

Bias	Low risk	Unclear	High risk
Sequence generation	44 (55%)	13 (16.25%)	23 (28.75%)
Baseline characteristics	39(48.75%)	4 (5%)	37 (46.25%)
Allocation concealment	2 (2.5%)	74 (92.5%)	4 (5%)
Random housing	50 (62.5%)	10 (12.5%)	20 (25%)
Blinding (assessment)	4 (5%)	70 (87.5%)	6 (7.5%)
Random outcome assessment	53 (66.25%)	9 (11.25%)	18 (22.5%)
Binding (outcome)	13 (16.25%)	65 (81.25%)	2 (2.5%)
Attrition bias	51 (63.75%)	12 (15%)	17 (21.25%)
Selective outcome reporting	1 (1.25%)	61 (76.25%)	18 (22.5%)
Other source of bias	28 (35%)	24 (30%)	28 (35%)

### Prosocial paradigms: Definitions

**Fig 4:**
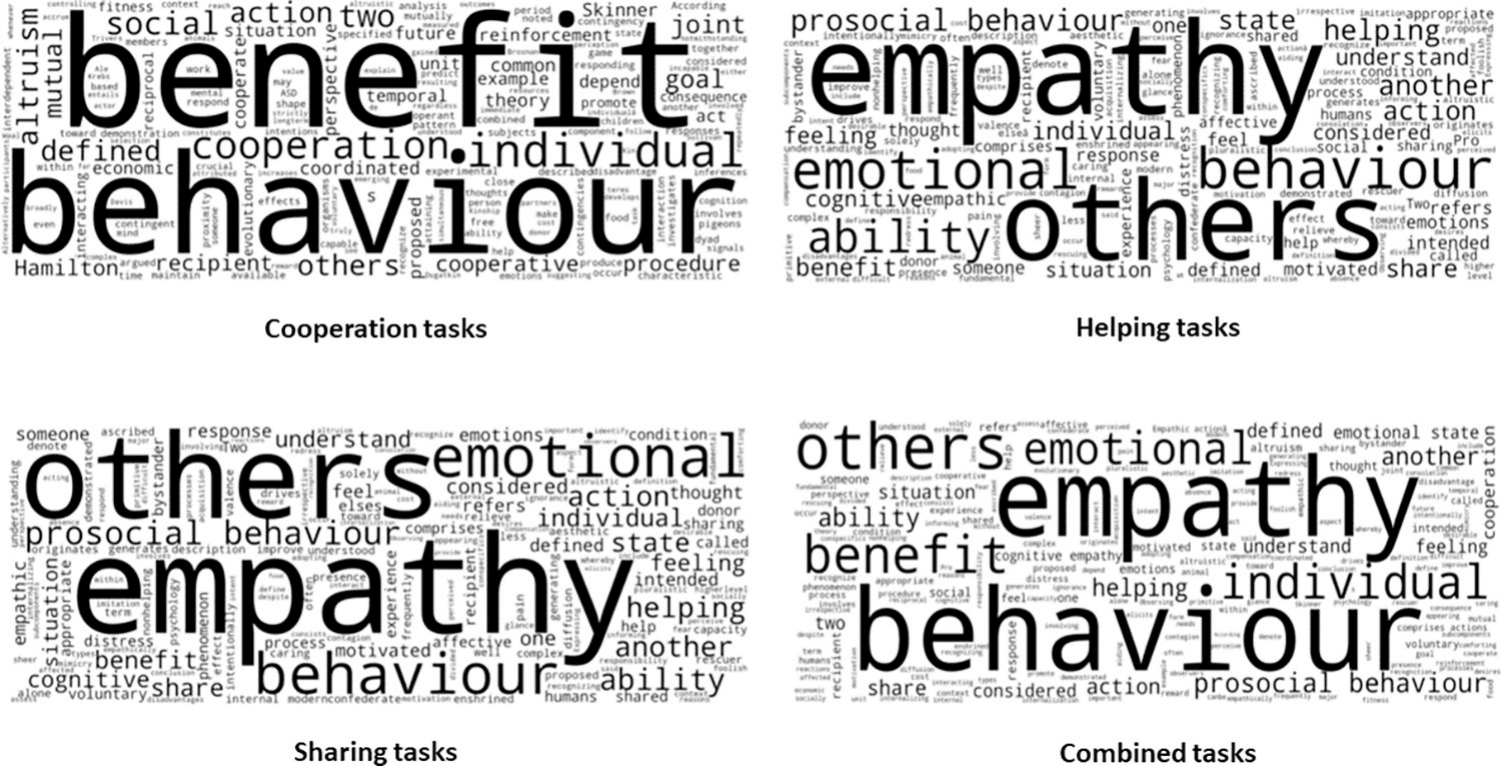
Word clouds of prosocial tasks–definition.

Of the 80 included articles, 69 provided a definition to describe the action of helping, sharing, or cooperating. Using word clouds, which is described as a collection of words depicted in different sizes according to the mode with more frequent terms being highlighted [118], a definition for each task (cooperation, helping and sharing) was extracted (Fig 4; see S2 Appendix for detailed definitions).

From the world clouds, the following definitions were extracted 1) Cooperation task: Combined or coordinated behavior of 2 or more individuals for mutual benefit or to benefit the recipient (with or without a disadvantage for the donor); 2) Helping task: Action that provides benefit or help to another, and 3) Sharing task: Task in which two or more individuals alternate in helping each other.

### Animals

In total, 7 articles reported using mice and 73 used rats. The selected mouse strain included C57BL/6 (5), Shank2 and 4 (1), and Balb-c mice (1). Regarding rats, strains included Sprague-Dawley (28), Wild type Norway (16), Long-Evans (11), Wistar (14), Lister Hooded (3), and a mix of strain (Long-Evans/Sprague-Dawley (3), and Long-Evans/Lister Hooded (1)). One study failed to specify the tested rat strain. On average, the included articles used 47 rodents. Six studies did not specify the sex or the included number of females and males. Forty-two studies used males only, 19 used females only, and 13 included both sexes. From all the articles including females, one study specified the animals to have been ovariectomized, while another specified doing vaginal smears after each experimental session. Both these studies assessed male and female rats. The majority of studies (*n* = 50) used adult rodents, 6 used adolescents, 2 used older rats and 1 study used a combination of adolescent and adult rats. Fifteen studies did not specify the age of the rodents and 37 studies failed to report the animals’ weight. Sixty-five studies mentioned maintaining ad libitum water intake, with 6 studies restricting water intake throughout the testing period and 9 omitting such mention. As for food restriction, 50 studies maintained ad libitum food intake, 22 mentioned restricted food intake (between 85 and 90% of free-feeding body weight), and 8 omitted this information. A majority of studies (*n* = 63) did not specify if the animals were littermates or unrelated, while 4 specified rodents to be unrelated and 13 mentioned animals to be littermates.

### Housing conditions

Housing conditions were the following: paired (*n* = 39), individual (*n* = 2), group housing ranging from 4 to 10 animals per cage (*n* = 34), a mix of single and paired housing (*n* = 3), and not specified (*n* = 2). Forty-four studies used a 12h light/dark cycle with lights on in the morning (i.e., experimental sessions conducted during light phase) while 27 used a reversed 12h light/dark cycle with lights on in the evening (i.e., experimental sessions conducted during the dark phase). Three studies used a reverse 14:10 light/dark cycle (i.e., testing sessions occurring during the dark phase), and 2 used a 16:8 light/dark cycle (i.e., testing sessions occurring during the light phase). Four studies did not specify the selected light/dark cycles. Although most studies (*n* = 70) were conducted using regular cage housing conditions, 10 studies used enriched environment housing [60, 73, 86–91, 110–113]. From these studies, 8 specified using cages enriched with paper and wood toys, a tunnel, a wooden shelter, or digging material, with some using a salt block. Two studies used two-story cages with the first floor containing running wheels, a shelter, woodblocks, a maze leading to a tube with food, and a ladder giving access to the second floor.

### Apparatus

Twenty-nine studies reported using a computerized apparatus such as an operant box, digital counter and/or computer-controlled levers or doors. The remaining articles (*n* = 51) used manually operated apparatus (e.g., maze, open field). Thirty-four studies recorded behaviors, with 9 also recording ultrasonic vocalizations. Seventy articles specified that animals could see, hear, and smell each other during the testing sessions.

### Type of reward/punishment

The articles using a cooperation task (*n* = 16) either provided a food reward (*n* = 9) such as food pellets, sucrose pellets or oat flakes, or a liquid reward (*n* = 7) such as liquid sucrose or water. Most studies selecting a helping task (*n* = 22) used social contact as a reward or a mix of food and social contact (*n* = 6). The remaining articles either used a mix of social contact as a reward and no social contact as a punishment (*n* = 1), heroin as a reward (*n* = 2), a mix of reward and punishment (in the form of food and foot shocks, *n* = 1), did not specify (*n* = 2) or did not use a reward (*n* = 3). All articles using a sharing task (*n* = 27) provided a food reward (e.g., food pellets, sucrose pellets, oat flakes, or pieces of banana).

### Duration of the task

Most of the included articles reported the duration of the experimental task in minutes (*n* = 60), with the average length being 21.64 minutes. The rest of the studies (*n* = 20) quantified the experimental sessions by reporting the number of trials, coordinated lever press or reward deliveries. Sixty-nine studies reported the total duration of the experiment (in days), which lasted 14.5 days on average. The remaining 11 studies did not report duration.

### Habituation and pretraining

In this review, habituation refers to the allotted time a rodent is placed in the novel environment with the possibility to freely explore it, while pretraining refers to the period allotted for rodents to learn specific actions (e.g., lever presses, nose poke, door opening). Of the 80 articles, 40 used a habituation period and 51 included a form of pretraining.

Of the 16 articles using a cooperation task, 6 used a habituation period ranging from 5 to 14 days, with a duration lasting from 10 to 30 minutes. Thirteen reported a form of pretraining consisting of learning how to climb a platform, press a lever, or roll a ball. All the studies used a specific criterion to determine when the pretraining was achieved (e.g., 25 responses per 20-min session).

Of the 37 articles using a helping task, 25 reported a period of habituation lasting between 2 to 14 days and ranging from 10 to 60 minutes. Fifteen articles reported a pretraining for which the duration to learn a specific action (i.e., door opening) varied from a single session (i.e., one day) up to 12 consecutive days (i.e., one session per day).

Of the 27 articles using a sharing task, 9 reported a period of habituation ranging from 1 to 2 days and lasting between 10 to 20 minutes. Twenty-three reported a pretraining period that was divided in two stages: (1) individual stage: initial exposure to learn how to pull/press/poke to obtain a reward, (2) paired stage: pairing the rodent with another conspecific to learn how to pull/press/poke to give a reward to the congener only. Most studies reported 11 days for individual training and 18 days for paired training.

### Control group

In total, 58 articles reported using a control, the most popular ones being an empty compartment or a toy condition.

### Experimental interventions

In total, 18 studies included an experimental manipulation using a specific drug, such as oxytocin (*n* = 5), heroin (*n* = 1), magnesium (*n* = 1), acetaminophen (*n* = 1), buprenorphine (*n* = 1), muscimol (*n* = 1), dimethyl sulfoxide (*n* = 1), honeybee venom (*n* = 1), 5-HT1A receptor agonist 8-OH-DPAT (*n* = 1). benzodiazepine (*n* = 2), AAV5-CamKIIa-mCherry Virus (*n* = 2) and Ibotenic acid (*n* = 1). Except for heroin, which was used as a reward, all studies used these drugs as an experimental variable.

Thirteen articles included an experimental manipulation requiring a surgery such as electrodes (*n* = 2), cannulas (*n* = 4), intravenous catheters (*n* = 2), head trauma (*n* = 1), lesion in the basolateral amygdala (*n* = 1), ovariectomy (*n* = 1), viral brain injection (*n* = 1), and microelectrode with a telemetry sensor (*n* = 1).

Thirty-three articles included other tests such as the open field (*n* = 14), the elevated plus maze (*n* = 6), the dominance tube test (*n* = 4), the forced swim test (*n* = 2), the magnitude discrimination task (*n* = 2), the preference test (*n* = 2), the Rotarod performance test (*n* = 2), the kin discrimination test (*n* = 1), the hole-board test (*n* = 1), the social interaction test (*n* = 2), the self-grooming test (*n* = 1), a test of memory capacity (*n* = 1), the boldness test (*n* = 4), water or food competition test (*n* = 2), social conditioned place preference (*n* = 3), affective state discrimination (*n* = 1), maternal deprivation (*n* = 2), and observational fear conditioning (*n* = 2).

### Performance index

Amongst the 27 articles using a sharing task, the most reported performance index was the total number of actions (e.g., stick pulling, lever presses, both-choice reward) enabling an animal to share with a congener. In the prosocial choice task (*n* = 9), all animals preferred the both-choice reward (giving a reward to the actor and partner rats) versus the individual-choice reward (only rewarding the actor). Two studies found that rats preferentially shared with a previously generous partner than a selfish one, suggesting a reciprocity effect [35, 113]. The other 18 studies using a sharing task (repeated donation game and sharing task using levers or sticks) used the frequency and latency of stick pulling or lever presses as the main performance index. Most studies (*n* = 17) found that rodents tended to perform more prosocial actions (share a food reward by pulling a stick or pressing a lever) to previously generous partners than defective ones.

The main performance index reported by studies using cooperation tasks was the number of successful coordinated actions (e.g., lever presses, maze running, nose poke, compartment entering). All studies using a prisoner’s dilemma (*n* = 5) found that rodents cooperated more often than they defected (i.e., animals pressed on the cooperation lever more often than the defection lever), and 2 reported reduced likelihood for food restricted rats to cooperate compared to ad *libitum* fed congeners [98, 99]. They also tended to cooperate more with previously cooperating partners than defecting ones. All studies using a cooperation learning task (*n* = 11) found that rodents could learn and perform a coordinated task to receive a reward, and 2 identified visual cues as crucial to the success of coordinated behaviors [52, 25].

The main performance indexes for helping tasks were the frequency and latency of rescuing behavior (i.e., door opening). The majority of studies reported that rodents could learn to open a door to rescue a congener. The frequency of door openings significantly decreased in control conditions (i.e., empty restrainer, ball of yarn, toy). Four studies also used a food condition, allowing a rodent to open the restrainer door containing a food reward [17, 32, 96, 116]. Two of these studies reported that the food condition had the shortest latencies of door-opening [32, 96], and one study reported that rats would open the door of the restrainer containing the conspecific prior to opening the one containing the food reward [17]. Additionally, one study reported similar latencies in selecting social release or food [116]. Finally, one study also supported light-intensity exercise to increase release behavior [114].

### Behavioral analyses

In total, 29 studies included behavioral analyses in their results. Studies using helping tasks most frequently reported behavioral analyses (*n* = 13) compared to sharing (*n* = 5), and cooperation tasks (*n* = 3).

In the helping paradigms, studies showed that rodents tended to stay close to the restrainer/soaked area while the trapped conspecific was inside, and then tended to enter the empty compartment after the freeing. Authors suggested that this behavior could be explained by a desire for social contact, which is often considered a reward in rescuing tasks [17, 53, 55, 70, 24, 96]. One study found that rats preferentially opted to play with a free conspecific (e.g., social opportunity) rather than liberate a trapped congener in an adjacent compartment [109]. Six studies reported distress/alarm calls (22 kHz) through ultrasonic vocalizations in restrained rats [17, 70]. One study reported no influence of enriched versus standard housing on emitted alarm calls [111]. One study also reported free rats to only emit alarm calls when a conspecific was placed in the restrainer, and not during the control condition (i.e., cotton ball in the restrainer) [115]. Finally, a study reported increased alarm calls from the target rat when their partner reduced helping behavior [107]. Other behavioral analyses revealed maternal care in early life to be associated with an ability to respond faster to a distressed conspecific [103]. In contrast, early life maternal deprivation was associated with a reduced motivation to free a trapped cagemate, although this result did not reach significance [111]. These two studies suggest early life experiences to play a role in regulating rodents’ prosociality.

Studies using a sharing task showed that rodents tended to modify their behaviors according to the partner’s action. For example, rats showed increased allogrooming and food donations to previously generous partners [88]. They also appeared sensitive to the food-seeking and social investigation of the recipient, which increased the prosocial actions [87]. A study using a direct reciprocity task revealed that rats pulled rewards differently for cooperators versus non-cooperators depending on the partner’s aggressive behavior (e.g., aggressivity towards non-cooperators tended to increase sharing behaviors) [60]. Similarly, Gachomba and colleagues [30] found social hierarchy to contribute to prosocial choices. They found that dominant rats would acquire prosocial tendencies faster and more often selected the altruistic option compared to submissive counterparts. Scheggia and colleagues [31] also found dominant mice to show a preference for altruistic choices in a sharing task, while subordinate mice preferentially selected selfish actions. Two studies investigated the importance of sensory information. One study reported visual cues to have no impact on the latency of stick pulling, suggesting that visual information exchange is not required [60]. Another study supported the role of olfactory cues showing increased willingness of a rat to donate food when olfactory information from the partner was available [65]. Moreover, one study found that the number of non-prosocial male rats in an enriched environment was higher than rats in a standard housing, although large individual differences were observed amongst the animals [73]. Ultrasonic vocalizations recorded by one study revealed calls were more frequent when the actor failed to produce the sharing behavior to draw the partner’s attention towards the reward (50 kHz) [87].

Finally, studies using a cooperation task showed a tendency for animals to synchronize their behaviors with their partners, such as waiting for the conspecific before performing an action (nose poke, platform climbing) [56, 74]. All studies (*n* = 3) also assessed behavioral profiles using the social dominance test. Two used the prisoner’s dilemma and reported no effect of dominance status on pellets received or responses made [67, 99]. One study found that a natural hierarchy seemed to be installed amongst paired rats, in which one would become the leader of the dyad (e.g., the first to climb onto the platform) [105]. They also reported significantly reduced anxiety levels in the leader compared to the follower rat [105]. Finally, one study recorded ultrasonic vocalizations and reported that rodents produced ‘happy’ calls during cooperative behaviors and increased social interactions (50 kHz) [25].

### Sex differences

Of the 80 included articles, 17 used both sexes and 8 amongst them investigated sex differences. Four studies did not find differences between males and females regarding task performance (i.e., freeing task, prisoner’s dilemma, sharing task) [73, 99, 101, 107]. Of the four studies that did find a sex difference, one reported elevated activity levels in females compared to male rats during a cooperation task, which could explain improved performance in the task (i.e., coordinating behavior with a congener in a maze to receive a reward) [52]. Another study observed a tendency for female rats to open the door and free a congener more frequently than males. However, this study used significantly less females (*n* = 6) than males (*n* = 24) [17]. Amongst the two studies using a sharing task, one reported female mice to show increased sharing toward familiar versus unfamiliar conspecifics [33], while the other one identified males as being more prosocial than female rats [31]. Finally, although the authors reported no sex differences in door opening, a study found that females emitted more alarm calls than male rats during a helping task [107].

### Age differences

Only one study tested possible effects of rodents’ age by comparing responses of adolescent and adult rats in a helping task [29]. Findings indicated that adolescent rats released a trapped conspecific more consistently and quicker than adult counterparts. Moreover, adult rats selectively released ingroup rather than outgroup members, which was not the case for adolescent rats. This could potentially suggest that filiation (ingroup bias) develops over time to impact behavioral responses in adulthood [29].

### Strain differences and familiarity

Of the 80 studies, 4 assessed strain differences and 3 investigated the impact of familiarity. One study assessed the strain familiarity of Sprague-Dawley rats in a helping paradigm (door opening) using Sprague-Dawley and Long-Evans rats, both in a familiar or unfamiliar (i.e., stranger rat) condition. The study showed that Sprague-Dawley rats did not open the door to free Long Evans strangers. Rats also tended to help strangers from their own strain more often than strangers of a different strain, which suggests an in-strain bias [53]. Similarly, a study also indicated a propensity for rats to preferentially release conspecific of the same strain, supporting familiarity as a factor influencing prosociality [104]. Another study evaluated Wistar rats with different genotypes (Wild-Type and Heterozygous) in a repeated donation game. The authors observed no differences between the genotypes regarding task performance (i.e., number of pushes to share a food reward) [63]. Regarding familiarity, one study using a sharing task showed no impact of familiarity on rats’ sharing behaviors [30]. A study assessing strain differences in Lister Hooded versus Long Evans rats in a cooperation learning task found that Long Evans took longer (i.e., in days) to complete the initial phase of the training (i.e., nose poke) compared to Lister Hooded rats. However, the slope of the learning curve was similar for both strains after this initial phase [74]. Finally, a study assessed two different types of mice (Shank2 and Shank3) in an autism spectrum disorder model, in which mice had to coordinate their running through a maze to receive a reward. The findings support Shank3 mice to display attenuated social cooperative behavior and reduced activity compared to Shank2 mice [67].

## Discussion

This scoping review extracted and analyzed 80 articles with the aim of better characterizing prosocial paradigms in rodents, identifying potential gaps in reporting, and exploring mediating factors of prosociality. Initially, 59 articles published between 2000 and 2020 were included. An updated search conducted by semi-supervised machine algorithm included 21 articles published between 2021 and 2023. Results were the following: (1) Three categories of tasks were extracted: cooperation, helping, and sharing tasks, (2) Rodents performed prosocial actions in all three categories, (3) Significant gaps in reported methodologies (e.g., animals’ characteristics, housing conditions, and/or experimental protocol) and mediating factors (e.g., sex, age, strain, housing) were identified, and (4) The incorporation of behavioral analyses provided valuable and pertinent insights into the prosocial behavior of rodents, complementing performance-based metrics. Nevertheless, numerous studies neglected to incorporate such analyses.

### 1. Prosociality in rodents: Three categories of tasks

**Fig 5:**
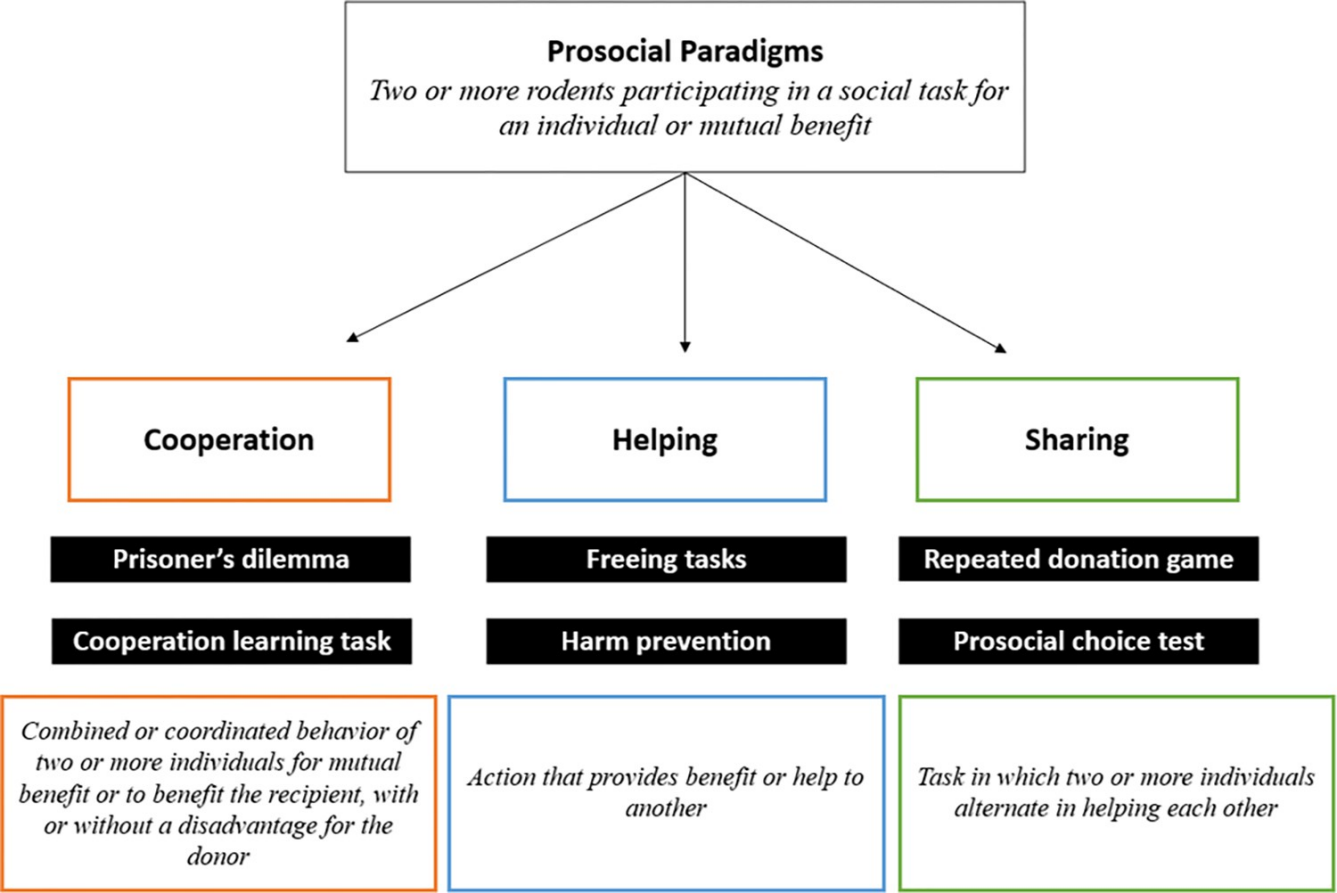
Prosocial paradigms: Proposed definitions of related tasks.

While prosocial tasks have been diversified and broadly defined [119] the results of this review consistently noted three distinct tasks and their related definitions (cooperation, helping, and sharing tasks; see Fig 5).

### Cooperation tasks

The definition for cooperation tasks (*combined or coordinated behaviors of two or more individuals for mutual benefit or to benefit the recipient*, *with or without a disadvantage for the donor*) highlights the importance of coordinated behaviors for the pursuit of benefit (i.e., for one recipient or both). The cooperation tasks involved two rodents that were trained to give a response (e.g., lever pressing, running through a maze) within a set time interval to obtain a mutual reward. Another variation involved the prisoner’s dilemma, in which both rodents decided to either cooperate with mutual benefits or defect by pressing on a lever within a certain time interval to receive a ‘selfish’ reward.

This review found cooperation tasks to be the least commonly used prosocial paradigms, being selected by 20% of the screened articles (16/80). This is likely related to the extensive pretraining periods. Amongst screened studies, 81% (13/16) performed pretraining tasks requiring rodents to reach a specific criterion (i.e., 8 pulls within 7 minutes, 30 rewards in 10 minutes, pressing the lever ≥100 times/session for two consecutive days) prior to the experimental sessions. These training sessions required substantive investments (i.e., time and costs), with studies reporting pretraining sessions lasting from 18 to 20 days with a specific criterion needed to be reached by each rodent [85, 25]. While it is known that animals learn at a different pace [120–122], setting a specific criterion to be reached prior to moving onto the next stage ensures that all animals understand the task and its associated rewards [123, 124]. In this context, cooperation tasks tend to be considered of higher complexity in terms of required cognitive resources [80], hence rendering a pretraining period essential when using these types of paradigms. A drawback of extensive pretraining is related to some rodents taking much longer than others to learn, with some never reaching the set goal and being excluded from the study, which might create a reporting bias [125, 126].

### Helping tasks

The helping paradigm was the most popular in this review, representing 46% of the total number of included studies (37/80). The popularity of this paradigm could be explained by the well-disseminated 2011 research findings by Ben-Ami Bartal and colleagues [17], in which rats opened a door to rescue a trapped congener. This study can be considered a pioneer in the assessment of helping behaviors in rodents, as all the articles included in this review were published subsequently. The definition for helping tasks (*action that provides benefit or help to another*) refers to a specific action that a rodent can do (or not) to explicitly benefit (e.g., giving a reward) or help (e.g., freeing from a restrainer) a congener. Specifically, helping tasks involves pressing a lever to free a congener trapped in a restraining device or a soaked area, or to prevent an electric shock to a congener (harm prevention). All studies using a helping paradigm included an aversive component (e.g., restraining device, soaked area or foot shock). This could be explained by the idea that aversive models trigger distress in animals, which is more explicitly defined and easily observed in rodents (e.g., freezing behavior) than behaviors emulating positive affect [21].

Of all the included studies, 68% of studies (25/37) using a helping paradigm used a habituation period and 40% (15/37) included a form of pretraining. The main goal of pretraining was to teach rodents to open the restrainer door, by either pulling/pressing a lever or breaking through a paper lid. Despite the minimal inclusion of pretraining sessions, the majority of studies reported the willingness of rodents to open the door to rescue a congener. This interesting finding could be influenced by the aversive/distressing component of the task (i.e., being restrained or trapped in a soaked area) possibly recruiting the rat’s survival instinct as a motivation to free a congener. This is concordant with findings from studies on fear contagion in rodents which found that when one conspecific is showing signs of distress (e.g., alarm calls, freezing behaviors) the other, although only witnessing the distressing situation, quickly displays the same fearful signs [12, 127]. This fear contagion allows rodents to respond appropriately to environmental threats [8].

### Sharing tasks

The definition for sharing tasks (*tasks in which two or more individuals help each other in turn*) puts an emphasis on alternate actions to benefit one another. A commonly used sharing task involved two rodents that could press a lever or pull a stick to give each other food rewards. Another variation entailed a rodent having the option to consume a food reward in a solitary compartment or select an alternative compartment for a mutual reward (i.e., a prosocial choice task). Unlike cooperation tasks, sharing paradigms do not require coordinated behavioral responses between the two animals.

Twenty-seven studies (33.75%) used a sharing paradigm. Interestingly, this category was the only one to exclusively use non-aversive tasks. While sharing tasks had the lowest rate of habituation period (33%, 9/27), it also had the highest rate of pretraining sessions (85%, 23/27). Most studies opted for a pretraining in two stages: (1) An initial stage where the rodent would learn how to pull/press/poke to obtain a reward, (2) An ensuing paired stage where the rat learned to pull/press/poke to give a reward to a conspecific. This pretraining in two steps allows the animal to (1) familiarize itself with the rewards (e.g., food pellet, sucrose water), (2) learn the levers’ contingency, and (3) associate lever presses with donation to a congener. Pretraining seems essential, particularly for tasks that involve sharing, due to their inherent complexity. Indeed, rodents must learn the contingency of the presented options (e.g., levers, sticks) and associate this response with the impact on the congener (e.g., food sharing). This is consistent with previous research demonstrating the critical importance of pretraining for sharing tasks (Charron et al., 2022).

### 2. Rodents demonstrated prosociality in all three categories

Across all three task categories, rodents demonstrated the capacity to engage in prosocial behaviors. This finding indicates that all three tasks are suitable options for exploring prosocial behaviors in rats and mice, as they consistently yield meaningful results, as evidenced by the performance indexes.

All studies using a cooperation task supported rodents’ ability to perform a coordinated action with a congener to obtain a mutual reward. Studies also found that rodents tended to cooperate more with previously cooperating partners than defecting ones. This indicates that rodents can remember previously cooperative partners and their intention to cooperate. This phenomenon called direct reciprocity is manifested through rodents adjusting the quality (e.g., giving the opportunity to share more palatable food rewards like bananas versus foods with lower levels of attractiveness such as carrots) of their help depending on the partner’s previous help [61]. From an evolutionary perspective, this behavioral response matters for survival as a rodent is more susceptible to help a previously collaborative congener based on the premises that future help can be expected [80].

All studies using a helping task found rodents to be able to learn to rescue a congener. Acquisition of this competence is supported by the different control conditions, where the latency of door openings significantly increased when rodents had the possibility to ‘rescue’ a control (e.g., toy rat, a ball of yarn or open an empty restrainer) indicating that rodents can differentiate between a distressed congener and a control condition.

Similarly to cooperation tasks, studies using a direct reciprocity task reported that rats showed increased prosocial behaviors (e.g., sharing a food reward) to previously cooperative versus defective partners. This finding is consistent with observations of increased help reciprocity within rodent dyads using previously generous versus defecting partners. This supports the ability of rodents to recognize and remember helpful partners and the importance of such recognition in guiding prosocial responses, exemplified through increased allogrooming and food donations to previously sharing partners [79]. Rats also appeared sensitive to the food-seeking and social investigation of the recipient rat, a factor increasing the occurrences of prosocial actions.

### 3. Gaps in reporting and mediating factors of prosociality

This scoping review revealed many gaps in reported methodologies as revealed by the risk of bias assessment. For cooperation tasks, the highest risk found amongst the articles was the report on baseline characteristics: 61% of studies (8/13) were identified with a high risk. A high risk of bias was attributed if the baseline of the animals and groups was not comparable without any rationale or methodological explanation for this approach. Studies failed to address the age difference between the tested groups (e.g., animals of varying age), selection of rodents with superior performance during pretraining, had varied housing conditions (e.g., with up to 5 rats housed per cage) and/or failed to identify and discuss previous exposure of some rodents to cognitive tests. Defining a study protocol prior to starting the experiment could have prevented these methodological issues [128]. While certain issues may be challenging to resolve due to logistical constraints or limited access to resources, it is crucial to acknowledge and address the potential impact of these choices to promote transparency and facilitate replication [128].

Regarding helping tasks, the highest risk of bias found amongst studies was the other sources of bias (46%, 17/37). Studies failed to report the results of tests previously conducted, mentioned cleaning testing apparatuses with a wet sponge (this being insufficient to remove previously tested rodent’s odors) [129], and failed to report methodological information regarding the coding of video recordings (e.g., manually, with software, how many researchers coded) and inter-coder reliability scores. Ideally, coders should be blind to the conditions and inter-coder reliability should always be assessed [130].

Sharing tasks showed the most elevated occurrence of ‘high risk’ flags. Many studies failed to report the methodology with sufficient details enabling replication (e.g., failed to specify the rats’ age or housing conditions, left a portion of the social task procedure unexplained, provided no information on the habituation or handling process, did not indicate the inter-coder reliability score or only used one coder, or failed to randomly assign the experimental groups and/or housing conditions). It is strongly recommended that authors follow the ARRIVE guidelines when designing animal studies, as well as using a guide for reporting animal studies, such as the one published by Grundy [131].

### Impact of mediating factors on prosocial behaviors

Extracted data from the 80 included articles revealed potential mediating factors of prosociality in all 3 categories of tasks, including housing, aggression and dominance, strain and familiarity, food restriction, and sex differences. Although each factor did not include enough studies to draw strong conclusions, highlighting these trends may inform future studies investigating the expression of prosocial behaviors in rodents.

### Housing

While many studies reported using an enriched environment (*n* = 10), only two investigated its potential impact on prosociality. One study reported environmental enrichment (EE) to be associated with an increased number of ‘non-prosocial’ male rats compared to standard housing, although large individual differences were observed amongst the animals [73]. Interestingly, the other study found that maternal separation impaired mutual reward preference in a prosocial choice task, but that exposure to an enriched environment prevented this impairment [110]. Although EE conditions can vary considerably between studies [132], it has been associated with beneficial effects on brain plasticity, cognition, and improved mental and physical health [34, 133]. Such housing conditions also promote animal welfare [133, 134]. Although limited, observations show that EE might have an impact on the expression of prosociality in rodents and that additional studies are needed.

### Aggression and dominance

The impact of aggression and dominance was investigated by studies using cooperation and sharing tasks only. Of the 16 studies using cooperation tasks, 2 investigated dominance (using the dominance tube test). They both reported no effects of the dominance status on cooperative behaviors in male mice and rats, and in ovariectomized female rats [67, 99]. These results contrast findings from other studies showing dominant rats to display increased motivation for food rewards [135], or a correlation between dominance and social motivation [136]. This difference might be explained by females that were ovariectomized during the adolescence stage, which could have impacted results of the study [99]. Pubertal hormones could play a role in modulating aggression and dominance in female rodents [137], and pubertal ovariectomy is known to impact brain development [138], including preventing the development of social recognition abilities [139], impairments in spatial learning [140], and social investigation [141]. In contrast to cooperation tasks, 3 out of 27 studies using a sharing task found an impact of dominance and hierarchy on sharing behavior [30, 31]. Gachomba and colleagues [30] reported that dominant rats tended to acquire prosocial tendencies faster than submissive conspecifics. Behavioral analyses also revealed that submissive animals tended to increase proximity towards the focal (donor) rat and would follow them around the choice area, while dominant animals would respond to these cues by increasing their attention to the recipient through increased sniffing around the submissive animal [30]. Similarly, Scheggia and colleagues [31] found that prosocial actions were directed more frequently towards dominant actors, and that selfish decisions were more often made by submissive than dominant mice. Finally, one study found that rats pulled levers differently depending on the partner’s aggressive behaviors. The authors suggested that cooperators can reduce helping behavior by showing aggression whereas non-cooperators might increase helping behaviors of their partners by being aggressive towards them [60]. Interestingly this study was the only one allowing physical contact in the apparatus. It is known that rats and mice establish a social hierarchy [142], and the possibility for physical contact increases the chances of aggressive and dominance behaviors [143]. As demonstrated in this review, aggression and dominance behaviors seem to have a mediating impact on prosociality in rodents and remain an important element for future studies to document.

### Food restriction

Four studies investigated the impact of food restriction on prosocial behaviors. Three studies using a helping task included a food condition, where a rodent could open a restrainer containing food. Two of these studies found that the food condition (i.e., restrainer containing a food reward) had the shortest latency of door-opening [32, 96] whereas one study found that rats would open the door of the restrainer containing the conspecific first followed by the restrainer containing the food [17]. Interestingly, the two first studies used a food restriction paradigm while the third one used ad libitum fed rats. This finding is similar to the cooperation paradigm, in which studies found that ad libitum fed rodents cooperated more than food deprived ones [98, 99]. Food restriction in rodents can alter impulsiveness and the incentive value of food rewards [98, 144], which could explain the increased willingness to cooperate in sated rats. Rodents are commonly maintained between 80–85% of free-feeding body weights to maximize motivation to participate and facilitate operant responding [99, 145, 146]. However, some have raised welfare concerns of such practice [145, 146], arguing that the individual food intake and adult body weight can significantly vary among animals [145] and that such practice does not account for strain, age, housing conditions, exercise opportunities, and so forth [145–147]. Finally, the circadian rhythm is known to regulate calorie intake, with rodents consuming 70 to 85% of their diet during the dark phase of the circadian cycle [145, 148]. Thus, the period at which restricted feeding is imposed is also important to consider, as it can influence daily food/calorie intake, and consequently impact task performance.

## Strain and familiarity

Of the 7 studies investigating strain differences and the impact of familiarity on prosocial behaviors, 4 found performance differences amongst different strains and levels of familiarity. For example, rats seem to present an in-strain bias, with Sprague-Dawley not opening the door to free Long-Evans strangers [53]. Moreover, Sprague-Dawley tended to help strangers from their own strain, but not from a different one [53]. Another study supported the preference for rats to release conspecific of the same strain [104]. Interestingly, findings from Breton and colleagues [29] found that ingroup and outgroup bias seem to emerge during adulthood, with adolescent rats sharing as much with ingroup and outgroup members, but not adult rats. These findings suggest that the choice of strain, the age of the rodents, and the level of familiarity between the animals can play a role in the expression of prosocial behaviors and more research is needed to provide further insight in this mediating factor of prosociality.

### Sex differences

Of the 80 included studies, 42 only used males (52.5%), 19 used only females (23.75%), 13 included both sexes (16.25%), and 6 did not specify (7.5%). These study samples are consistent with underrepresentation of females in the scientific literature. In neuroscience, only 20% of studies include both sexes, and for every research on females, there are five publications with males [149, 150]. One of the reasons mentioned for this discrepancy is related to the menstrual/estrous cycle and hormonal fluctuations, which could create variability amongst females [150]. The role of hormones such as estrogen, estradiol, and progesterone on the regulation of cognitive processes (e.g., memory, learning, decision-making) in females is well documented, particularly in relation to the abundance of receptors that respond to these hormones in the hypothalamus, pituitary gland, hippocampus, and prefrontal cortex [151, 152]. To date, sex-related differences have been widely studied in relation to hormonal secretion, which regulates different aspects of social behaviors, including social interactions, social learning, reproduction, and aggression [153, 154]. In this review, only one study specified doing vaginal smears after each experimental session to determine the levels of circulating sex hormones throughout the estrous cycle [73]. Interestingly, they found that females did not show an overall preference for the prosocial option regardless of their estrous cycle [73]. Mate choice also requires cognitive functions of social recognition and vicarious social learning, two behaviors partly regulated by estrogen secretion in females [153, 154]. Indeed, ovariectomized females show reduced social recognition, whereas the injection of estrogen alone or in combination with progesterone restores this cognitive function [155]. In this review, one study specified that females have been ovariectomized to control for hormone levels [99]. The authors found no sex differences regarding direct reciprocity. To date, examination of sex-related differences in rodents’ prosociality remains preliminary and needs further investigation and replication [28]. As highlighted, not enough studies have investigated sex differences to enable clear conclusions on the effect of sex on prosocial behaviors.

### 4. Behaviors are determinant in rodents prosociality

Results from this scoping review revealed behavioral analyses during prosocial testing to provide a source for important refinement in the interpretation of collected observations. However, many included articles failed to report such analyses. When performed, behavioral analyses included video recording of the task and audio recording of ultrasonic vocalizations.

### Behavioral analyses

Of the 37 studies using a helping task, 15 also investigated behavioral patterns. In general, authors found that rodents tended to stay close to the restrainer or soaked area while the trapped conspecific was inside. They also displayed a tendency to enter the compartment once the congener was free, indicating a potential desire for social contact. This behavior could serve as a reward in this context, implying that the underlying motivation may be social interaction rather than the intention to help another [24, 53, 66, 97]. Interestingly, 5 articles prevented social contact from happening after the freeing action, and all showed that rodents could also learn to open the door [18, 70–72, 102]. These contrasting findings suggest that social contact might not be the only motivation driving rodents’ helping behaviors. Hence, further research is warranted to more precisely define the influence of social contact on helping tasks. Regarding cooperation tasks, Conde-Moro and colleagues [56] used a paradigm in which rats had to simultaneously climb a platform to receive a reward. They found that the ‘leader’ rats would initiate climbing onto a first platform and wait for their partner to reach the same level prior climbing the second platform. This clearly demonstrates a specific behavioral pattern that took place during the cooperation task. Similarly, Kozma and colleagues [74] indicated rats to keep close body contact with their partner, possibly to influence or control the partner’s action. These results show that analyzing rodents’ behaviors can give access to a rich array of information on prosociality [27, 148]. Consequently, researchers strictly limiting analyses to task performance might miss important data stemming from rodents’ behavioral patterns [156, 157].

### Ultrasonic vocalizations

Rodents communicate with each other using frequencies undetectable to the human ear [133]. Such communication can be investigated through the recording of ultrasonic vocalizations (USVs). It is known that rats tend to emit low frequency ‘alarm’ calls (∽22 kHz) when in distress [133] and high frequency ‘happy’ calls during play and mating [158]. Only one study amongst the 16 using a cooperation task performed such analyses. They found prosocial behavior to be positively correlated with the number of emitted ‘happy calls’ (∽50 kHz). The calls were recorded before and after a simultaneous nose-poke response, suggesting that the task might have been considered reinforcing (appetitive) for the rats [25]. While all studies using a helping task included an aversive component, only two recorded USVs [70, 115]. Aversive paradigms tend to trigger alarm calls, which alert the other conspecific and can motivate its action (i.e., opening a door) [159]. Indeed, Hernandez-Lallement and colleagues [70] found that rats would produce an alarm call when trapped or receiving electric shocks, probably to inform the free rat of its distress [159]. Similarly, Subdhadeep and colleagues [115] found that trapped rats would emit distress and ‘happy’ calls before and after liberation, respectively. This suggests that communication through USVs could be at the core of the rodents’ motivation to open the door. Finally, one study using a sharing paradigm measured USVs [87]. Similar to findings from helping tasks, alarm calls were more frequently emitted in situations where the actor rat failed to produce the awaited sharing behavior, or when the actor wanted to draw the partner’s attention to a shared reward. While the evidence remains limited, studies examining USVs within the three prosocial paradigms indicate that vocalizations play a pivotal role in the manifestation of prosocial behaviors.

## Conclusion

Results from this scoping review revealed four important findings. Firstly, 3 categories of prosocial paradigms were extracted and defined. Secondly, findings from the 80 included articles showed rodents to be able to display prosocial behaviors in all 3 categories of tasks. Thirdly, significant gaps in reported methodologies, such as variations in animal characteristics, housing conditions, and experimental protocols, were identified, alongside potential mediating factors like sex, housing, aggression, dominance, strain, familiarity, age, and food restriction. Lastly, behavioral analyses unveiled significant behavioral patterns exhibited by rodents, which could potentially influence the expression of prosociality. While these findings demonstrate the ability for rats and mice to display prosocial behaviors in three categories of paradigm, this scoping review also informs future studies on the importance of behavioral analyses and mediating factors in the expression of prosociality in rodents. However, a limitation in studying prosocial behavior in rodents arises from the fact that many paradigms involve mutual benefits, such as the focal rodent also receiving a reward (either food or social interaction). This raises questions about the genuine motivation underlying prosocial behavior (e.g., are rodents engaging in tasks solely for a food reward or out of a desire to assist or benefit a congener?). While some reviewed studies have addressed this issue by restricting social contact or modulating food reward, further research is necessary to deepen our understanding of prosocial mechanisms in rodents. Consequently, integrating behavioral analyses alongside performance-based metrics for each task, as well as investigating further potential mediating factors involved in the expression of prosocial behaviors could be the next step to gain further insight into rodent prosociality.

## Supporting information

S1 Checklist PRISMA 2020 checklist. (DOCX)

S1 Appendix Supplementary file–complete search strategy. (DOCX)

S2 Appendix Supplementary data. All extracted data from the 80 included articles.
(DOCX)
