# Supplementary material for: In search of prosociality in rodents: A scoping review
Source: PLoS One. 2024 Nov 7;19(11):e0310771. doi: 10.1371/journal.pone.0310771 (PMC11542798; doi:10.1371/journal.pone.0310771)
Supplement: S1 Appendix — (DOCX) [file pone.0310771.s002.docx]

**Appendix 1. Supplementary File – Complete Search Strategy**

The following databases were initially searched on January 26, 2021:

- APA PsycInfo (Ovid) – 3920 results
- Embase (Ovid) – 6759 results
- MEDLINE (Ovid) – 5745 results
- Scopus – 3948 results
- Web of Science (Clarivate) – 4113 results

The following strategies were used for each database.

**APA PsycInfo (Ovid)**

1. rodents/
2. mice/
3. exp rats/
4. rodent*.ti,ab
5. (mouse or mice or "mus musculus").ti,ab
6. (rat or rats or rattus).ti,ab
7. or/1-6
8. empathy/
9. sympathy/
10. kindness/
11. altruism/
12. "sharing (social behavior)"/
13. exp animal emotions/
14. animal social behavior/
15. animal cooperation/
16. empathy.ti,ab
17. sympathy.ti,ab
18. (prosocial* or "pro social*").ti,ab
19. (compassion* or altru* or kindness).ti,ab
20. ((share* or sharing or help* or assist* or care* or caring or emotion* or empath* or sympath*) adj3 behav*).ti,ab
21. or/8-20
22. 7 and 21
23. limit 22 to yr="2000 -Current"

**Embase (Ovid)**

1. rodent/
2. exp mouse/
3. exp rat/
4. rodent*.ti,ab
5. (mouse or mice or "mus musculus").ti,ab
6. (rat or rats or rattus).ti,ab
7. or/1-6
8. empathy/
9. altruism/
10. social behavior/
11. care behavior/
12. cooperation/
13. empathy.ti,ab
14. sympathy.ti,ab
15. (prosocial* or "pro social*").ti,ab
16. (compassion* or altru* or kindness).ti,ab
17. ((share* or sharing or help* or assist* or care* or caring or emotion* or empath* or sympath*) adj3 behav*).ti,ab
18. or/8-17
19. 7 and 18
20. limit 19 to yr="2000 -Current"
21. 20 not human/

**MEDLINE (Ovid)**

1. rodentia/
2. exp mice/
3. exp rats/
4. rodent*.ti,ab
5. (mouse or mice or "mus musculus").ti,ab
6. (rat or rats or rattus).ti,ab
7. or/1-6
8. empathy/
9. altruism/
10. social behavior/
11. cooperative behavior/
12. helping behavior/
13. empathy.ti,ab
14. sympathy.ti,ab
15. (prosocial* or "pro social*").ti,ab
16. (compassion* or altru* or kindness).ti,ab
17. ((share* or sharing or help* or assist* or care* or caring or emotion* or empath* or sympath*) adj3 behav*).ti,ab
18. or/8-17
19. 7 and 18
20. limit 19 to yr="2000 -Current"
21. 20 not humans/

**Scopus**

1. TITLE-ABS-KEY(rodent*)
2. TITLE-ABS-KEY(mouse or mice or "mus musculus")
3. TITLE-ABS-KEY(rat or rats or rattus)
4. #1 OR #2 OR #3
5. TITLE-ABS-KEY(empathy)
6. TITLE-ABS-KEY(sympathy)
7. TITLE-ABS-KEY(prosocial* or "pro social*")
8. TITLE-ABS-KEY(compassion* or altru* or kindness)
9. TITLE-ABS-KEY((share* or sharing or help* or assist* or care* or caring or emotion* or empath* or sympath*) w/3 behav*)
10. #5 OR #6 OR #7 OR #8 OR #9
11. #4 AND #10
12. PUBYEAR > 1999
13. #11 AND #12

**Web of Science (Clarivate)**

1. TS=(rodent*)
2. TS=(mouse or mice or "mus musculus")
3. TS=(rat or rats or rattus)
4. #1 OR #2 OR #3
5. TS=(empathy)
6. TS=(sympathy)
7. TS=(prosocial* or "pro social*")
8. TS=(compassion* or altru* or kindness)
9. TS=((share* or sharing or help* or assist* or care* or caring or emotion* or empath* or sympath*) near/3 behav*)
10. #5 OR #6 OR #7 OR #8 OR #9
11. #4 AND #10
12. PY=(2000-2021)
13. #11 AND #12
