# Supplementary material for: In search of prosociality in rodents: A scoping review
Source: PLoS One. 2024 Nov 7;19(11):e0310771. doi: 10.1371/journal.pone.0310771 (PMC11542798; doi:10.1371/journal.pone.0310771)
Supplement: S2 Appendix — All extracted data from the 80 included articles. (DOCX) [file pone.0310771.s003.docx]

**Appendix 2 - Original search – Characteristics of the included studies (2000-2020)**

**Table 1.** Results of all Included Studies – Animals.

| **Reference** | **Type of rodent** | **#rodents** | **#males** | **#females** | **Age** | **Strain** | **Weight (g)** | **Water restriction** | **Food restriction** |
| --- | --- | --- | --- | --- | --- | --- | --- | --- | --- |
| Avital et al., 2016 | Rats | 40 | 20 | 20 | NA | Wistar | 240-260 | Yes | No |
| Bartal et al., 2011 | Rats | 97 | 85 | 12 | 3-6 months | Sprague Dawley | 270g-380 | No | No |
| Bartal et al., 2014 | Rats | 59 | 59 | NA | 8-11 weeks | Sprague Dawley + Long-Evans | NA | No | No |
| Bartal et al., 2016 | Rats | 154 | 154 | NA | 2 months | Sprague Dawley | NA | No | No |
| Blystad et al., 2019 | Rats | 30 | NA | 30 | 100pnd | Sprague Dawley | 150-200 | No | Yes |
| Carvalheiro et al., 2019 | Rats | 18 | 18 | NA | 41 to 53pnd | Wistar | NA | No | No |
| Conde-Moro et al., 2019 | Rats | 12 | 12 | NA | 3 months old | Lister Hooded | 250-300 | No | Yes |
| Cox et al., 2020 | Rats | 102 | 102 | NA | NA | Sprague-Dawley | 250-275 | No | Yes |
| Daghestani et al., 2017 | Rats | 60 | NA | NA | PND7 | Sprague Dawley | NA | No | No |
| de Carvalho et al., 2018 | Rats | 20 | 20 | NA | exp1: 3 to 6 months, exp2: 3 months | Wistar | NA | Yes | No |
| Delmas et al., 2019 | Rats | 30 | 30 | NA | 2 months | Long-Evans | 300-330 | No | Yes |
| Dolivo & Taborsky 2015 (A) | Rats | 43 | NA | 43 | NA | Wild-type Norway | NA | No | No |
| Dolivo & Taborsky 2015 (B) | Rats | 20 | NA | 20 | 22 months | Wild-type Norway | NA | No | No |
| Donovan et al., 2020 | Rats | 80 | 80 | NA | 6 weeks | Long-Evans | 200 | No | No |
| Festucci et al., 2020 | Rats | 8 | 8 | NA | at least 4 months | Wistar-Han DAT knockout | 300-400 | NA | NA |
| Fontes-Dutra et al., 2019 | Rats | 50 | 50 | NA | 69-81 days experimental sessions | Wistar | NA | NA | NA |
| Gerber et al., 2020 | Rats | 115 | NA | 115 | NA | Wild type Norway | 300 | No | No |
| Hachiga et al,. 2018 | Rats | 18 | 18 | NA | 2 weeks | Sprague Dawley | NA | NA | NA |
| Han et al., 2020 | Mice | NA | unknown | NA | 8-12 week | KO and WT littermates | NA | Yes | NA |
| Havlik et al., 2020 | Rats | 56 | 56 | NA | 8-11 weeks | Sprague Dawley and Long Evans | NA | No | No |
| Hernandez-Lallement et al., 2015 | Rats | 68 | 68 | NA | NA | Long-Evans | NA | No | Yes |
| Hernandez-Lallement et al., 2016 | Rats | 34 | 36 | NA | adult | Long-Evans | 250-450 | No | Yes |
| Hernandez-Lallement et al., 2020 | Rats | 314 | 302 | 12 | 30 days | Sprague Dawley | M:302.4;F:240.8 | No | Yes |
| Hosgorler et al., 2020 | Rats | 55 | NA | 55 | 3-6 months | Sprague Dawley | 200-250 | No | No |
| Kandis et al., 2018 | Rats | 32 | 32 | NA | Adult | Sprague Dawley | NA | No | No |
| Karakilic et al., 2018 | Rats | 30 | 30 | NA | Adult | Sprague Dawley | NA | No | No |
| Kentrop et al., 2020 | Rats | 160 | 124 | 36 | 8–10 weeks | Wistar | NA | No | Yes |
| Kozma et al., 2019 | Rats | 44 | 44 | NA | 1.5 y/o (LE) and 1 y/o (LH) | Long-Evans and Lister Hooded | LE:401-514; LH:377-556 | NA | Yes |
| Li & Wood, 2017 | Rats | 26 | 26 | NA | Adult | Long-Evans | 200 | No | Yes |
| Lopuch & Popik, 2011 | Rats | 20 | 20 | NA | NA | Sprague Dawley | 225-250 | No | No |
| Marquez et al., 2015 | Rats | 74 | 74 | NA | NA | Sprague-Dawley | 375-425 | No | No |
| Oberliessen et al., 2016 | Rats | 23 | 23 | NA | 4-5 months | Long-Evans | 400-533 | No | Yes |
| Raz, 2013 | Rats | 81 | 81 | NA | pnd 49 | Wistar | 190 | No | No |
| Rutte & Taborsky, 2007 | Rats | 36 | NA | 36 | NA | Wild-type Norway | NA | No | No |
| Rutte & Taborsky, 2008 | Rats | 23 | NA | 23 | NA | Wild type | NA | No | No |
| Sato et al., 2015 | Rats | 56 | 46 | 10 | 10 weeks | Sprague-Dawley | EXP1 (214-f, 362-m), EXP2: 350, EXP3: 291 | No | No |
| Schmid et al., 2017 | Rats | 36 | NA | 36 | NA | Wild-type Norway | NA | NA | NA |
| Schneeberger et al., 2012 | Rats | 14 | NA | 14 | NA | Wild-type Norway | NA | No | Yes |
| Schonfeld et al., 2020 | Rats | 48 | NA | NA | 10 weeks | NA | NA | NA | Yes |
| Schwartz et al., 2017 | Rats | 29 | 20 | 9 | 15 weeks. Exp 6: 10 months | Sprague Dawley | NA | No | No |
| Schweinfurth & Taborsky, 2016 | Rats | 48 | NA | 48 | Adult | Wild-type Norway | NA | NA | NA |
| Schweinfurth & Taborsky, 2017 | Rats | 40 | NA | 40 | 1 y/o | Wild type Norway | 300 | No | No |
| Schweinfurth & Taborsky, 2018 (A) | Rats | 50 | NA | 50 | 2 y/o | Norway rats | 300g to 400 | No | Yes |
| Schweinfurth & Taborsky, 2018 (B) | Rats | 74 | NA | 74 | adult | Wild-type Norway | avg 300 | No | No |
| Schweinfurth & Taborsky, 2018 (C) | Rats | 21 | 21 | NA | 19 months | Wild-type Norway | 607 | NA | NA |
| Schweinfurth & al., 2019 | Rats | 41 | 23 | NA | adult | Wild-type Norway | Median of 627 | No | No |
| Schweinfurth & Taborsky, 2020 | Rats | 54 | NA | 54 | adult | Wild-type Norway | 350 | NA | NA |
| Silberberg et al., 2014 | Rats | 12 | NA | 12 | 3-6 months | Sprague Dawley | NA | No | No |
| Silva et al., 2020 | Rats | 52 | 52 | NA | 3-4 months | Wistar | NA | No | No |
| Tomek et al., 2019 | Rats | 64 | 64 | NA | NA | Sprague Dawley | 250 | Yes | Yes |
| Tomek et al., 2020 | Rats | 99 | 99 | NA | NA | Sprague-Dawley | 250 | No | No |
| Tsoory et al., 2012 | Rats | 27 | 27 | NA | 7-8 weeks | Wistar | NA | Yes | No |
| Ueno et al., 2019 (A) | Mice | NA | NA | NA | 10 weeks | C57BL/6N | NA | No | No |
| Ueno et al., 2019 (B) | Mice | 40 | 40 | NA | 10 weeks | C57BL/6N | NA | No | No |
| Viana et al., 2010 | Rats | 12 | 12 | NA | NA | Sprague Dawley | NA | No | Yes |
| Wood et al., 2016 | Rats | 48 | 32 | 16 | 6 weeks | Long-Evans | 200 | No | Yes |
| Yamagishi et al., 2020 (A) | Rats | 72 | 72 | NA | 7 or 11 weeks | Sprague Dawley | 314.62g | No | No |
| Yamagishi et al., 2020 (B) | Rats | 82 | 70 | 12 | EXP1: 11-weeks, EXP2: 12 weeks | Sprague Dawley | Exp 1: M: 370.64 (335-401); F: 244.92 (227-269g range); Exp 2 (M only): 398.35 (range 351-512g) | No | No |
| Yuksel et al., 2019 | Mice | 32 | 16 | 16 | Adult | Balb-c | NA | No | No |

**Table 2** **.**Results of all Included Studies – Housing Conditions and Apparatus.

| **Reference** | **Housing (per cage)** | **Relationship** | **Light-Dark Cycle** | **Enrichment** | **Operant Paradigm** | **Video/vocalization recording** | **Can animals see/hear/smell each other?** | |
| --- | --- | --- | --- | --- | --- | --- | --- | --- |
| Avital et al., 2016 | 3-4 | NA | 12:12 dl lights on @ 7am | No | Cooperation learning task | yes (video, ethovision) | Yes | |
| Bartal et al., 2011 | 2 | NA | 12:12 dl | No | Freeing task (tube) | Yes (video, ultrasonic voc) | Yes | |
| Bartal et al., 2014 | 2 | NA | 12:12 dl | No | Freeing task (tube) | Yes (video and voc) | Yes | |
| Bartal et al., 2016 | 2 | NA | 12:12 dl | No | Freeing task (tube) | No | Yes (+ touch) | |
| Blystad et al., 2019 | 2 | NA | 12:12 dl lights on @ 7 | No | Freeing task (tube) | Yes (video, ethovision) | | Yes |
| Carvalheiro et al., 2019 | 2 | Littermates | 12:12 dl | No | Freeing task (tube) | Yes (video) | Yes | |
| Conde-Moro et al., 2019 | 2 | NA | 12:12 dl | No | Cooperation learning task | Yes (video) | Yes | |
| Cox et al., 2020 | 2 | NA | R12:12 dl lights on @ 6pm | No | Freeing task (soaked area) | No | Yes (smell) | |
| Daghestani et al., 2017 | 10 | Split litter design | 12:12 dl | No | Freeing task (tube) | No | Yes | |
| de Carvalho et al., 2018 | 2 | NA | 12:12 dl | No | Cooperation learning task | No | Yes (see) | |
| Delmas et al., 2019 | 1-2 | NA | 12:12 ld lights @ 9am | No | Prisoner's dilemma | No | Yes (see/smell -white noise machine preventing hearing) | |
| Dolivo & Taborsky 2015 (A) | 5 | Related (housing); unrelated for task | R12:12 dl lights on @ 20h00 | Yes | Repeated donation game | No | See/hear/smell in visual contact but not see specifically for the blind test | |
| Dolivo & Taborsky 2015 (B) | 5 | Related (housing); unrelated for task | R 12:12 ld lights on @ 20:00 | No | Repeated donation game | No | Yes | |
| Donovan et al., 2020 | 2 | NA | R14L:10D photoperiod | No | Prisoner's dilemma | No | Yes | |
| Festucci et al., 2020 | 2 | NA | NA | No | Repeated donation game - Box ('open field') | No | Yes | |
| Fontes-Dutra et al., 2019 | NA | Littermates | 12:12 dl | No | Freeing task (tube) | No | Yes | |
| Gerber et al., 2020 | 5 | Littermates | 12:12dl | No | Repeated donation game | No | Yes | |
| Hachiga et al,. 2018 | 3 | NA | 12:12 dl lights @ 8am | No | Freeing task (tube) | No | Yes | |
| Han et al., 2020 | 2-4 | Littermates | NA | No | Cooperation learning task | Yes (video, ethovision) | Yes | |
| Havlik et al., 2020 | 2 | NA | 12:12ld lights on 6am | No | Freeing task (tube) | Yes (video) | Yes | |
| Hernandez-Lallement et al., 2015 | 4 | NA | NA | No | Prosocial choice task - Double T Maze | No | Yes | |
| Hernandez-Lallement et al., 2016 | 3 | NA | R12:12 dl | No | Prosocial choice task - Double T Maze | No | Yes (hear/smell) | |
| Hernandez-Lallement et al., 2020 | 4 | NA | R12:12 lights OFF @ 7am | No | Not harming - Operant box | Yes (video and sound) | Yes | |
| Hosgorler et al., 2020 | 2 | NA | NA | No | Freeing task (soaked area) | Yes (video, ethovision) | NA | |
| Kandis et al., 2018 | 2 | NA | 12:12 dl | No | Freeing task (soaked area) | No | Yes | |
| Karakilic et al., 2018 | 2 | NA | 12:12 dl | No | Freeing task (soaked area) | No | NA | |
| Kentrop et al., 2020 | 6-10 | NA | 12:12 ld lights on @ 8am | Yes | Sharing task (lever presses) | No | Yes | |
| Kozma et al., 2019 | 3 | NA | R12:12 ld lights on @ 17h | No | Cooperation learning task (nose poke) | No | Yes (+ touch) | |
| Li & Wood, 2017 | 2 | NA | R14L:10D photoperiod | No | Repeated donation game | No | Yes | |
| Lopuch & Popik, 2011 | 4 | NA | 12:12 dl lights on @ 7am | No | Cooperation learning task (nose poke) | Yes (video and ultrasound voc) | Yes + touch (except in phase 2) | |
| Marquez et al., 2015 | 2 | NA | R12:12 ld lights OFF @ 10 am | No | Prosocial choice task - Double T Maze | Yes (video) | Yes | |
| Oberliessen et al., 2016 | 2-3 | Non-related | R12:12 ld lights off @ 7am | No | Prosocial choice task - Double T Maze | No | Yes | |
| Raz, 2013 | 5-6 | NA | 12:12 dl | No | Cooperation learning task | Yes (video, ethovision) | Yes | |
| Rutte & Taborsky, 2007 | 3-7 | Littermates | R12:12 lights on @ 20h | No | Lever presses - Box ('open field') | No | Yes | |
| Rutte & Taborsky, 2008 | 3-7 | Littermates | R12:12 ld lights on @ 8pm | No | Repeated donation game | Yes (video) | Yes | |
| Sato et al., 2015 | 2 | Non-related | 16:8 ld lights on @ 8 am | No | Freeing task (soaked area) | No | Yes | |
| Schmid et al., 2017 | 3-6 | NA | R12:12 dl | No |  | Yes (video) | Yes | |
| Schneeberger et al., 2012 | 3-5 | Related (housing); unrelated for task | R12:12 ld lights on @ 8 pm | No | Repeated donation game | No | Yes | |
| Schonfeld et al., 2020 | 3 | NA | R12:12ld | No | Prosocial choice task - Double T Maze | No | Yes | |
| Schwartz et al., 2017 | 3 | NA | 12:12 ld lights @ 8am | No | Freeing task (soaked area) | No | NA | |
| Schweinfurth & Taborsky, 2016 | 3-5 | Related (housing); unrelated for task | Reversed 12:12 ld lights on @8pm | No | Repeated donation game | No | Yes | |
| Schweinfurth & Taborsky, 2017 | 5 | Littermates | R12:12 dl lights on @ 8pm | Yes | Repeated donation game | No | Yes | |
| Schweinfurth & Taborsky, 2018 (A) | 3-5 | Littermates | R12:12 ld lights on @ 8pm | Yes | Repeated donation game | Yes (video, ultrasonic voc) | Yes | |
| Schweinfurth & Taborsky, 2018 (B) | 3-5 | Littermates | R12:12 ld lights on @ 8pm | Yes | Repeated donation game | No | Yes | |
| Schweinfurth & Taborsky, 2018 (C) | 4 | Non-related | R12:12 ld lights on @ 8pm | No | Sharing task (stick pulling) | No | Yes | |
| Schweinfurth & al., 2019 | 4 | NA | R12:12 lights on @ 20h | Yes | Repeated donation game | No | Yes | |
| Schweinfurth & Taborsky, 2020 | 1-5 | Related (housing); unrelated for task | R12:12 ld lights on @ 8pm | Yes | Prisoner's dilemma | No | Yes | |
| Silberberg et al., 2014 | 2 | NA | 12:12 dl | No | Freeing task (soaked area) | Yes (video) | Yes | |
| Silva et al., 2020 | 2 | NA | 12:12 dl | No | Freeing task (tube) | Yes (video) | Yes | |
| Tomek et al., 2019 | 2 | NA | R12:12 ld lights @ 7pm light off at 7am; 22= reversed 12:12dl, | No | Freeing task (tube) | No | Yes | |
| Tomek et al., 2020 | 2 | NA | R12:12 ld light on @ 7am | No | Freeing task (tube) | Yes (video) | Yes | |
| Tsoory et al., 2012 | 2-5 | NA | 12:12 ld lights on @ 7am | No | Cooperation learning task | No | NA | |
| Ueno et al., 2019 (A) | 5 | NA | 12:12 ld lights on @ 8am | No | Freeing task (tube) | Yes (video) | Yes (see) | |
| Ueno et al., 2019 (B) | 5 | NA | 12:12 ld | No | Freeing task (tube) | No | Yes | |
| Viana et al., 2010 | 2 | Non-related | 12:12 dl | No | Prisoner's dilemma in a double T-Maze | No | Yes (see/smell) | |
| Wood et al., 2016 | 2 | NA | R14:10 dl | No | Prisoner's dilemma in operant box | No | Yes | |
| Yamagishi et al., 2020 (A) | 1-2 | NA | 16:8 dl | No | Freeing task (soaked area) | Yes (video) | Yes (see) | |
| Yamagishi et al., 2020 (B) | 2 | NA | 12:12 dl | No | Freeing task (soaked area) | Yes (video) | Yes (see) | |
| Yuksel et al., 2019 | 2 | NA | 12:12 dl | No | Freeing task (soaked area) | No | NA | |

**Table 3.** Results of all Included Studies – Methodology.

| **Reference** | **Duration (in min)** | **# of Testing Days** | **Habituation** | **Pretraining** | **Reward/Punishment** | **Control Group** | **Other Behavioral Tests** | **Drugs or Surgical Interventions** |
| --- | --- | --- | --- | --- | --- | --- | --- | --- |
| Avital et al., 2016 | 15 | 24 | Yes | No | 70ul of sucrose water solution (20%) | Yes | NA | NA |
| Bartal et al., 2011 | 40-60 | 128 | Yes | No | Social Reward/chocolate chips | Yes | NA | NA |
| Bartal et al., 2014 | 40-60 | 12 | Yes | No | Social reward | No | Open field | NA |
| Bartal et al., 2016 | 40-60 | 12 | Yes | No | Social reward/chocolate chips | No | Open field | MDZ (2mg & 1.25mg/kg, i.p.), nadolol (10mg/kg) or saline |
| Blystad et al., 2019 | 5-10 | 15 | Yes | Yes | Food Pellets/social reward | Yes | Light vs dark envi. | NA |
| Carvalheiro et al., 2019 | 60 | 12 | Yes | No | dark box/social reward | Yes | NA | NA |
| Conde-Moro et al., 2019 | 40presses | until reached criterion | Yes | Yes | Pellets | Yes | NA | Rats implanted with 2 sets of recording electrodes at the right prelimbic cortex |
| Cox et al., 2020 | 5 | 5 | No | No | social contact/ no social contact | Yes | NA | NA |
| Daghestani et al., 2017 | 30 | 3 | No | No | Social reward | Yes | Hole-board test, social contact test, open field, self-grooming test | BV (subcutaneous 0.5 mg/kg/ day for 31 days)  PPA (oral, 250-mg/kg/day body weight for 3 days) |
| de Carvalho et al., 2018 | 45-75 water deliveries | 10 sessions | No | Yes | Water | Yes | NA | NA |
| Delmas et al., 2019 | 45sec | until reached criterion | No | Yes | Sugar pellets | NA | NA | NA |
| Dolivo & Taborsky 2015 (A) | NA | 18 sessions | No | Yes | food pellet | Yes | NA | NA |
| Dolivo & Taborsky 2015 (B) | 7 | 4 | No | Yes | Piece of banana or carrot, cereal flake | NA | NA | NA |
| Donovan et al., 2020 | 24 trials | 10 sessions | No | Yes | 45mg sucrose pellet | Yes | NA | i.p. of saline or Oxytocin (0.1 mg/kg) |
| Festucci et al., 2020 | NA | 12 | No | Yes | 2g of pellets | Yes | NA | NA |
| Fontes-Dutra et al., 2019 | 40 | 12 | Yes | No | social reward | Yes | NA | injections of RSV, VPA, RSV+VPA or saline |
| Gerber et al., 2020 | 14 | NA | No | Yes | 1 oat flake | Yes | NA | NA |
| Hachiga et al,. 2018 | 3 | 24 sessions | No | Yes | social reward | NA | NA | NA |
| Han et al., 2020 | 15 | 12 | Yes | No | 70 ul 20% sucrose | NA | Dominance test | NA |
| Havlik et al., 2020 | 40 | 12 | Yes | No | Social reward | Yes | Open field, elevated plus maze | benzodiazepine (2 mg/kg i.p. increased at 4) |
| Hernandez-Lallement et al., 2015 | 8/10forced+15free choice trials | 40 | Yes | Yes | 3 pellets | Yes | NA | NA |
| Hernandez-Lallement et al., 2016 | 6forced + 25free choice trials | 12 | Yes | Yes | 3 sucrose pellets | Yes | Magnitude discrimination task | 22 actors had surgery in BLA |
| Hernandez-Lallement et al., 2020 | 4forced+20free choice trials | NA | Yes | No | Pellets + foot shocks | Yes | NA | Muscimol or saline, cannulas in ACC |
| Hosgorler et al., 2020 | 5 | 12 | No | No | NA | Yes | Rotarod test, plus maze, open field, forced swim test | Magnesium sulphate (30 mg/kg intramuscularly); Magnesium citrate (27 mg/kg perorally by gavage); Magnesium acetyl laurate (50 mg/kg perorally by gavage)+Head trauma |
| Kandis et al., 2018 | 5 | 12 | Yes | No | NA | Yes | Open field, Elevated plus maze, Rotarod performance test | Acetaminophen once a day orally for 11 days (100, 200, 400mg) |
| Karakilic et al., 2018 | 1 | 12 | Yes | No | Social reward | Yes | Open field, elevated plus maze | NA |
| Kentrop et al., 2020 | 30 | 19 (f) 30 (m) | Yes | No | Sucrose pellets | No | Boldness test | NA |
| Kozma et al., 2019 | 20 | until reached criterion | No | Yes | Sucrose pellet | Yes | NA | NA |
| Li & Wood, 2017 | 72trials | 10 days per condition | No | Yes | Sucrose pellet | Yes | NA | NA |
| Lopuch & Popik, 2011 | NA | 44 | No | Yes | liquid sucrose (10ul; sucrose solution 20%) | No | NA | NA |
| Marquez et al., 2015 | 40 | 8 days on average | Yes | Yes | Sucrose pellet | Yes | NA | NA |
| Oberliessen et al., 2016 | NA | 24 | Yes | Yes | sucrose pellet | Yes | Hierarchy assessment | NA |
| Raz, 2013 | 30 | 4 | Yes | No | 0.5ml sucrose solution (10%) | No | Open field, sucrose preference test, acoustic startle response, two-way active shuttle avoidance | NA |
| Rutte & Taborsky, 2007 | 7 | 17 | No | Yes | oat flake | Yes | NA | NA |
| Rutte & Taborsky, 2008 | 7 | 20 | No | Yes | 1 oat flake | No | NA | NA |
| Sato et al., 2015 | 5 | 66 | No | Yes | 6 pieces of chocolate cereal (Kellogg's Japan Chocowa) | Yes | Preference test | NA |
| Schmid et al., 2017 | 7 | 6 | No | Yes | Raisins halves | Yes | NA | NA |
| Schneeberger et al., 2012 | 14 | NA | No | Yes | 1 oat flake | Yes | NA | NA |
| Schonfeld et al., 2020 | 6forced+15free choice trials | NA | Yes | No | 3 sucrose pellets | Yes | Open field, Magnitude discrimination task | Daily injections of 50 ng of the 5-HT1A receptor agonist 8-OH-DPAT or Ringer solution;cannulas targeting BLA |
| Schwartz et al., 2017 | 3 | 40 sessions | No | No | Social contact | Yes | NA | NA |
| Schweinfurth & Taborsky, 2016 | 14 | NA | No | Yes | Oat flake | Yes | NA | NA |
| Schweinfurth & Taborsky, 2017 | 14 | NA | No | Yes | 1 oat flake | Yes | NA | NA |
| Schweinfurth & Taborsky, 2018 (A) | 14 | NA | No | Yes | Piece of banana | No | NA | NA |
| Schweinfurth & Taborsky, 2018 (B) | 20+7 | NA | No | Yes | 1 oat flake | Yes | NA | NA |
| Schweinfurth & Taborsky, 2018 (C) | 14 | NA | No | Yes | 1 oat flake | Yes | Kin discrimination test | NA |
| Schweinfurth & al., 2019 | 7 | 20 | No | Yes | Oat flake | No | NA | NA |
| Schweinfurth & Taborsky, 2020 | 7 | 10 | No | Yes | Oat flake | Yes | Test of memory capacity | NA |
| Silberberg et al., 2014 | 30 | 57 daily sessions | Yes | No | Freeing/social contact | Yes | NA | NA |
| Silva et al., 2020 | 30 | 12 days per condition | Yes | No | Social reward | No | NA | NA |
| Tomek et al., 2019 | 60-30 | Phase1: 14 days, Phase 2:10-14 days, Phase 3: 3 days | Yes | No | social reward, heroin, and sucrose | No | NA | Heroin (0.06mg/kg), intravenous catheters in jugular vein |
| Tomek et al., 2020 | 60-30 | 24 | Yes | No | Social reward | Yes | NA | buprenorphine (0.05 mg/kg), control virus (AAV8-CaMKIIα-EGFP), DREADD virus (AAV8-CaMKIIα-hM3D(Gq), inhibitory DREADD virus (AAV8-CaMKIIα-hM4D(Gi), + Clozapine-N-Oxide (CNO) 1.5mg/kg i.p. injection); intravenous catheters in jugular vein |
| Tsoory et al., 2012 | 20 | 10 | Yes | Yes | 0.04 ml of a sweet saccharine solution [0.06%] | Yes | NA | NA |
| Ueno et al., 2019 (A) | 90 | 7 | No | Yes | Social reward or food reward | Yes | Social interaction test | NA |
| Ueno et al., 2019 (B) | 90 | 10 | No | Yes | Social reward | Yes | NA | Oxytocin (100 μg/ kg) |
| Viana et al., 2010 | 20 trials | 10 | No | Yes | Food pellets + tail pinches | Yes | NA | NA |
| Wood et al., 2016 | 25 trials | 4 | No | Yes | Sucrose pellets | Yes | Dominance test | Female rats were ovariectomized |
| Yamagishi et al., 2020 (A) | 10-15 | 4-20 | Yes | No | Social reward | No | NA | intraperitoneal injections oxytocin (1.0 mg/kg) |
| Yamagishi et al., 2020 (B) | 10 | 10 | Yes | No | Social reward | Yes | NA | 0.2 μl oxytocin receptor antagonist + cannula into ACC |
| Yuksel et al., 2019 | 5 | 1 | Yes | Yes | Escape water | Yes | Elevated plus maze, open field | NA |

**Table 4.** Result of all Included Studies - Performance and Behavioral Analyses.

| **Reference** | **Performance index** | **Behavioral Analyses** |
| --- | --- | --- |
| Avital et al., 2016 | # of coordinated behavior (learning and latent social coop. learning can be achieved; divider that enabled perception of sensory modalities ↑ coop. Enabling tactile perception led to poor performance while visual availability ↑ performance.) | NA |
| Bartal et al., 2011 | Frequency and latency of door openings (Rats were motivated to move and act specifically in presence of trapped rat. In trapped condition, the % of rats that opened the door ↑, and latency to door-opening ↓. Rats in chocolate condition shared in half the trials.) | Sig more alarm calls were recorded during trapped condition (13%) than during empty and object conditions (3 to 5%). |
| Bartal et al., 2014 | Frequency and latency of door openings (Most rats in both SD cagemate (6/8, 75%) and SD stranger (10/12, 83%) conditions became openers. Rats were as motivated to help strangers as they were to help cagemates.) | Openers spent more time around closed restrainer than non-openers. After freeing rats, sig less fights were observed for openers vs non-openers. Rats were less active before door-opening vs after. |
| Bartal et al., 2016 | Door opening latency (↓ over days in all groups indicating learning, except MDZ injected rats) | NA |
| Blystad et al., 2019 | # and latency of door openings (door opening was largely the same as when the restrainer was baited with food. Latency was shortest when restrainer contained food, intermediate when it contained a cagemate and longest when it was empty. Latencies for dark (89.9s) and light (82.7s.)) | NA |
| Carvalheiro et al., 2019 | # of door openings (prosocial behavior still occurred when given escape alternative. Rats that could escape showed sig fewer door openings (first opening within 8 days) and took more time to open the door than rats that could not escape (first opening within 2 days). All rats, except one, opened the door after 12 sessions.) | Trapped rat’s struggling behavior (associated with restraint stress) did not affect door-opening latency. ↑ exploratory behavior was predictive of faster door opening. |
| Conde-Moro et al., 2019 | # of coordinated press (rats synchronized their platform climbs sig more when paired than when individual.) | One rat (leader) was the first to climb onto the platform most of the times and seemed to wait on the platform for the partner (follower) to climb for each coop. trial. |
| Cox et al., 2020 | Chain pull latency (when removing social interaction rats still learned to release a distressed cage mate and retained the task for an extended period. When the distressed Target or Rtarget was either replaced or removed in 3-chamber task, chain pull latency of the Observers and R-observers sig ↑.) | NA |
| Daghestani et al., 2017 | # of door openings (Sig lower # of trials of PPA-treated rat pups to open the restrainers and help the encaged rat pups vs control. Other treated groups, particularly the BV-treated group, displayed a comparatively high pro-social behavior vs PPA-treated group. ) | NA |
| de Carvalho et al., 2018 | # of coop. responses (Coop. rates systematically ↑ as a function of ratio value for some of the dyads; for other dyads, responding remained constant or ↓ between.) | NA |
| Delmas et al., 2019 | # of coop. choices (High levels of coop. (86,11%) and mutual coop. (76,32%)) | NA |
| Dolivo & Taborsky 2015 (A) | # and latency of pulls (with no visual information exchange, test rats rewarded cooperators earlier than defectors.) | Rats ↓ help propensity by showing aggression, non-cooperators ↑ help propensity of partners by attacking them. |
| Dolivo & Taborsky 2015 (B) | Frequency of providing partner with reward (Rats adjust their help levels to the quality of help previously obtained. Rats pulled after a shorter delay for a cooperator who had provided them with preferred bananas than for one who had provided them with same amount of nonpreferred carrots.) | NA |
| Donovan et al., 2020 | # of coordinated lever presses (Tendency for Subjects to be nicer when their Stooge partner was also nice.) | NA |
| Festucci et al., 2020 | # of pushes (WT rats pushed regardless of reward. WT rats facing a WT partner pushed sig more than HET ones. When food pellet was used as reward, data for same genotype did not show sig differences.) | NA |
| Fontes-Dutra et al., 2019 | # of door openings (VPA animals are able to open restrainer, and continue to do so at the same frequency as controls. Rats of the VPA and VPA+RSV groups showed a delay in the expression of this helping behaviour, opening the restrainer for the first time on average 3 days later than controls.) | NA |
| Gerber et al., 2020 | # of food sharing (Rats donated food more often and earlier when olfactory information from their partner was available, irrespective of partner’s previous helpfulness.) | NA |
| Hachiga et al,. 2018 | # of goal box choices (Except for S4, all subjects preferred to go to rat locked in restraint tube more frequently when other box was empty (Cond.1) than when choice was between 2 rats, one in a tube and one free (Cond.2). All rats preferred to go to a rat outside restraint tube (Cond.3) than going to a rat locked in tube.) | NA |
| Han et al., 2020 | # of coordinated running (Shank2Δ6−7 and Shank3Δ9 mice displayed opposite behaviors in social dominance and coop. tests. Shank2Δ6−7 exhibited a trend (not sig) towards improved performance, in association with higher activity and elevated efficacy suggesting similar levels of motivated behavior between WT and Shank2Δ6−7. Shank3Δ9 exhibited ↓ social coop. behavior, a ↓ in the # of mutual rewards and reduced activity.) | Most male Shank2Δ6−7 exhibited frequent aggressive behaviors and ↑ anxiety level, likely accounting for the ↑ dominance in tube test. |
| Havlik et al., 2020 | Door opening latencies (Rats tested with incompetent helpers had fewer consecutive door-openings. SD rats familiar with LE strain tested with a LE incompetent helper resembled rats tested with one or two incompetent SD helpers in that they opened less frequently than controls. SD rats unfamiliar with LE behaved similarly to control.) | NA |
| Hernandez-Lallement et al., 2015 | # of BR choice (Actors have preference for BR compartment when paired with partner. Sig higher % of BR choices in partner vs toy condition. Negative correlation between normalized partner weight and SB scores.) | NA |
| Hernandez-Lallement et al., 2016 | NA | NA |
| Hernandez-Lallement et al., 2020 | # of lever presses (Many individual differences across rats, with only a subset showing strong switching. Actors that switched more delayed and shortened reward consumption following shocks to victim and oriented more toward the victim in shock trials. An actor’s prior experience with shocks ↑ switching. No effect of familiarity on harm aversion.) | USV of victim: pain squeaks + freezing was not associated with switching. Victims that ↓ time spent close to divider (due to shock-induced behavioral activity) were paired with actors with higher switching scores. |
| Hosgorler et al., 2020 | # of door openings (No sig difference between groups. On 3rd post-traumatic day, door opening time was sig longer in both trauma and Mg sulphate group than in control.) | NA |
| Kandis et al., 2018 | # of door openings (Reduced latency of freeing in all acetaminophen administrations (with 400mg being longer than 100 and 200mg.)) | NA |
| Karakilic et al., 2018 | # of door openings (the mean opening door latency ↓ in all animals. Low intensity stress group was quicker in opening door vs control. Low intensity acute stress improved empathic behavior. Higher intensity acute stress associated with less anxiety indicators.) | NA |
| Kentrop et al., 2020 | # of BR lever presses (Males had a preference for the option that yields a reward for both rats. Females did not show preference for prosocial option, regardless of estrous cycle. Rats in complex. housing did not show preference for pro-social lever pressing. Costly prosocial behavior ↓ pro-social choice.) | Complex housed rats were found to be either prosocial (52 %) or not prosocial (48 %), while the standard housed rats were prosocial (64 %), indifferent (18 %), or not prosocial (18 %) |
| Kozma et al., 2019 | # of nose poke (Analysis of the nose-poke latency data, sample video-recordings and the sig ↓ performance of rats in control experiment suggests real coop.) | Trained rats of both strains performed coop. though alternative ways. Both regular place exchanges of LE rats and close body contact of LH rats may have served to control action/presence of partner. |
| Li & Wood, 2017 | # of rewards (when partnered with cagemate in FV block, responses were consistent with reciprocal altruism. When paired with another partner (a stranger or good stooge), Responder response rates ↓. Ehen paired with good stooge, rats failed to adjust their Donor responses to obtain more pellets.) | NA |
| Lopuch & Popik, 2011 | # of nose poke (Coop. developed gradually as # of coop. responses ↑ during training. Coop. was ↓ by a partition restricting visual, acoustic, and physical communication but not by partition restricting only physical contact.) | Coop. was related to the # of 50 kHz USV “happy” calls and to intensity of social interactions. No difference in high frequency communication. |
| Marquez et al., 2015 | # of BR choice (Rats acquired preference for prosocial option, providing recipient with access to food-baited arm. Rats rapidly, but gradually, acquired preference for the prosocial side, possibly through learning the contingency between their choice and the outcome to the recipient.) | The # of social investigation bouts was similar across protocols, except for the ↑ # of social investigations in rats of ‘‘no display of preference’’ condition during time between focals’ decision and recipients’ retrieval of food. |
| Oberliessen et al., 2016 | # of BR rewards (Rats preferred equal outcomes more in social than in toy condition, although the effect was relatively small.) | NA |
| Raz, 2013 | # of reward obtained (Rats in social isolation received less rewards than those in partial isolation.) | NA |
| Rutte & Taborsky, 2007 | # of pulling (Rats that recently experienced help pulled more often than when they had not. Pulling frequency was on average 21% higher in helper treatment than in non helper treatment. Rats with previous experience of help pulled on average four times earlier.) | NA |
| Rutte & Taborsky, 2008 | # of stick pulling (Exp1: rats pulled more often for partner that had pulled for them in previous interactions than for a partner that had not. Exp 2: pulling frequency was higher for partner that had helped before than for a new partner after having received help from others.) | NA |
| Sato et al., 2015 | # of door openings (Exp1: 9/10 helpers showed door-opening behavior. In role-reversal sessions, all helper rats (soaked rats in previous sessions) exhibited door-opening behavior more rapidly than helper in door-opening sessions. Exp2: 1/8 helper showed opening behavior. Exp3: the # of sessions were not sig different between the two groups.) | NA |
| Schmid et al., 2017 | Pulling frequencies (Rats pulled sig more often for partners that had pulled for them before than for partners that had not.) | NA |
| Schneeberger et al., 2012 | # of stick pulling (Rats provided more help to cooperative than defective partners. The amount of help provided ↓ more strongly with ↑ costs when experimental partner was a defector. Hungry rats received more help for food if they were light, whereas if receiver was satiated, rats provided more help for heavy partners.) | NA |
| Schonfeld et al., 2020 | # of BR choices (There was neither a sig interaction effect between condition (partner vs. toy) and group (50 ng 8-OHDPAT, 25 ng 8-OH-DPAT or vehicle), nor sig main effects of condition or group on % BR-choices in learning phase. A sig interaction effect between condition and group on % BR-choices in the expression phase was found once BR-side assignment was fully learned.) | NA |
| Schwartz et al., 2017 | # of compartment entering with wet vs dry rat (Exp 1: Free rats preferred mingling with wet rat over dry rat in both conditions. Exp2: free rats chose goal box with wet rat on 0.66 of the trials during last 5 sessions. Exp5: Rats chose wet box on 0.72 of free-choice trials over last 5 sessions. Exp6: Free rat sig preferred trapped rat over empty goal box in initial and reversal conditions.) |  |
| Schweinfurth & Taborsky, 2016 | Frequency and latency of pulling (Rats distinguished between pulling for cooperator vs defector; they pulled sig less often for the latter. Rats pulled more often and earlier for cooperative partners than for an empty cage.) | NA |
| Schweinfurth & Taborsky, 2017 | # of stick pulling/pushing (Rats helped previously cooperative partners more often than previously defecting partners. Rats responded to the coop. of social partners and transferred experienced to different coop. task.) | NA |
| Schweinfurth & Taborsky, 2018 (A) | Frequency and latency of pulling (Hungry rats pulled more often for social partner than satiated ones and started earlier to do so. Regardless of whether focal rats were hungry or satiated, pulling rates were always lower for empty cage than for social partners.) | Rats showed food reaching behaviors: stretching paws or sniffing towards reward, vocalized in 50-kHz, 13/25 rats showed ‘attention-grabbing ‘ (noisy behaviors directed towards potential donor.) |
| Schweinfurth & Taborsky, 2018 (B) | # of stick pulling (Rats provided more help for previously cooperating than non-cooperating partners. Rats groomed previously cooperative food providers more often than non-cooperative ones and provided more food to previously cooperating high groomers than low.) | In response to ↑ allogrooming, 20 rats ↑ whereas 11 ↓ food provisioning. In response to receiving food, 21 ↑ whereas 11 ↓ allogrooming rate. |
| Schweinfurth & Taborsky, 2018 © | # of stick pulling (Rats helped unrelated partners more often than related ones. Rats provided less food to previously defecting partners than cooperating ones.) | NA |
| Schweinfurth & al., 2019 | # of food donations (Rats provided more food to previously experienced cooperators than defectors. Experiencing cooperating or defecting partners prior to providing food to an unknown partner did not alter rats’ donation rate.) | NA |
| Schweinfurth & Taborsky, 2020 | # of stick pulling (Rats donated food less often to partners that were defecting. Pulling rates were lower for partners that they had experienced for 4 days with conflicting coop. experiences than for partners that they only met cooperating/defecting once. Rats provided food more often to partners that had been coop. during last encounter prior to test than to partners that had been defecting during last encounter. ) | NA |
| Silberberg et al., 2014 | Latencies and frequencies of door openings (Exp 1: Latencies ↑ and response rate ↓ for all free rats. Exp 2: latencies ↓ for all free rats over sessions. Exp 3: touch-contact frequencies were higher for all rats than in Condition 1 even though between-condition contingencies were identical in the 2 conditions.) | 3/6 free rats spent most of their session time in contact with tube; 4/6 trapped rats spent most of their session time inside the tube after the rear door was opened. Most previously restrained rats returned for substantial periods of time to the tube, presumably to be next to free rats, which also spent much of their time in contact with the tube, presumably to be near the trapped rat. |
| Silva et al., 2020 | # of door openings (Exp 1: no sig results from phase 1 to 3, sig result between phase 1 and 4 being lower rates of opening in phase 1 than 4. Exp2: Phase 4 was sig higher than phase 1,2,3 Exp3: Phase 1 was sig higher than toy or empty box. Latency Opening: exp1 no sig, exp2 no sig in phase 1,2,3 but lower latency in phase 4. exp3 no sig diff between phases.) | Reduced social interaction in phase 3 (Random rat) than phase 1 and 2. |
| Tomek et al., 2019 | # of rescue rates (Rats with history of sucrose self-administration continue to rescue cagemate, while rats with history of heroin self-administration choose to continue heroin intake and not rescue cagemate.) | No observed evidence of sig heroin-induced stupor or other opioid-induced behaviors during test sessions. |
| Tomek et al., 2020 | # of door openings (History of heroin self-administration ↓ prosocial behaviors. Chemogenetic activation of AIC restored prosocial behaviors following heroin intake. No sig difference in heroin intake between animals receiving active or control virus, chemogenetic inhibition of the insula had no effect on prosocial behaviors or heroin intake.) | NA |
| Tsoory et al., 2012 | # of coordinated behavior (COOP rats learned to cooperate by coordinating their shuttles. COOP and IND rats did not differ in terms of individual shuttles and obtained reinforcements.) | NA |
| Ueno et al., 2019 (A) | # of door openings (Mice engaged in tube-opening behaviour to free conspecifics. Mice did not open paper lid of empty tubes. Mice showed tube-opening behaviour both to free cagemates and strangers. Hungry mice tended to open tube containing food before the conspecific. Mice freed conspecifics even at the cost of personal discomfort (wet floor). | Mice freed conspecifics and then entered the tube. Even in the absence of expression of distress or vocalisation by the conspecific, the mouse exhibited tube-opening behaviour. |
| Ueno et al., 2019 (B) | # of door openings (Mice did not open tube containing ball of yarn. No sig difference between latency to lid-opening with 1 and 2 cagemates. No sig difference in the latency to lid-opening between tubes placed close to and far from the constrained cagemate.) | NA |
| Viana et al., 2010 | # of cooperative choices (Rats cooperated more often than they defected. Food deprived rats defected more often. When playing against a reciprocating opponent, rats displayed behaviour composed of both coop. (reward trials) and alternating reciprocity (alternating temptation and sucker trials.)) | NA |
| Wood et al., 2016 | # of cooperative choices (Coop. was reduced under food restriction, rats made sig more operant responses but received fewer pellets.) | No effect of dominance status on responses made or pellets received. |
| Yamagishi et al., 2020 (A) | Latencies of door openings (Latency of door-opening in all groups across sessions. Rats in Solo Oxtgroup learned door-opening faster than PairOxt group.) | NA |
| Yamagishi et al., 2020 (B) | Latency of door openings (Exp 1: latency of door-opening in OTA group was longer than SLN group, latency in both OTA and SLN groups ↓ across sessions. Exp. 2: door opening latencies were no different in Early and Late groups in first session, but those in Early group were longer than Late group in last session.) |  |
| Yuksel et al., 2019 | # of door openings (Mean door-opening duration progressively ↓ with time in all groups. Exercised groups opened the door quicker than controls in empathy-learning period. Voluntary physical activity ↓ anxiety and ↑ empathy-like behavior in both males and females.) |  |

**Table 5.** Results of all Included Studies - Other Analyses.

| **Reference** | **Sex Differences** | **Strain Differences** |
| --- | --- | --- |
| Avital et al., 2016 | Females better in social coop. ↑ in mutual rewards rate observed in females is positively correlated with activity level. | NA |
| Bartal et al., 2011 | More females (6/6) than males (17/24) became door-openers. Females opened restrainer at shorter latency than males on days 7-12. Females were more active in trapped but not in empty condition. | NA |
| Festucci et al., 2020 | NA | No differences for the 2 genotypes |
| Kentrop et al., 2020 | Females did not show preference for BR side. | NA |
| Kozma et al., 2019 | NA | LE rats required sig more days to complete training than LH. LE in experienced- experienced pairs learnt faster than the other two. Naive-experienced pairs did not perform better than naive-naïve. |
| Wood et al., 2016 | For direct reciprocity, females were more likely than males to deliver food pellets to their partner, but both showed no sig preference to give pellets to their cagemate over unfamiliar same-sex partner. Responses of both sexes were reduced when tested with an unresponsive partner (bad stooge.) | NA |
| Yamagishi et al., 2020 (B) | No sex differences were found. | NA |

*Articles that had no reported information regarding results in this table were excluded from it*

**Table 6.** Results of all Included Studies – Reported definition.

| **Reference** | **Task** | **Reported definition** |
| --- | --- | --- |
| Avital et al., 2016 | Cooperation learning task | Social cooperation is defined as a joint action for mutual benefit1 that depends not only on the individual behavior but also on the behaviors of others. |
| Bartal et al., 2011 | Freeing task (tube) | Pro-social behavior refers to actions that are intended to benefit another. |
| Bartal et al., 2014 | Freeing task (tube) | Pro-social behavior comprises actions that improve the well-being of others |
| Bartal et al., 2016 | Freeing task (tube) | Helping refers to actions that intentionally benefit others. In humans, helping is often motivated by an empathic response to the distress and pain of others. |
| Blystad et al., 2019 | Freeing task (tube) | The term empathy originates from a description of feeling at one with aesthetic experience and was proposed to denote the feeling/understanding of the thoughts and behaviour of others |
| Carvalheiro et al., 2019 | Freeing task (tube) | Prosocial behavior refers to “voluntary actions that are intended to help or benefit another individual” such as helping an individual in need, sharing resources, and cooperating with others to achieve common goals. |
| Conde-Moro et al., 2019 | Cooperation learning task | According to game theory, cooperation is considered when two or more individuals work together toward a common goal |
| Cox et al., 2020 | Freeing task (soaked area) | Empathy can be defined as the capacity for shared emotional valence, which generates shared affective states and therefore drives behaviors most appropriate to the emotional condition of others |
| Daghestani et al., 2017 | Freeing task (tube) | NA |
| de Carvalho et al., 2018 | Cooperation learning task | The experimental analysis of cooperative behavior investigates the effects of consequences contingent on the combined or coordinated behavior of two or more individuals |
| Delmas et al., 2019 | Prisoner's dilemma | Altruism is a behavior by an individual that may be to his disadvantage but benefits others individuals. |
| Dolivo & Taborsky 2015 (A) | Repeated donation game | Altruistic behaviour, in the sense that an individual performs a costly act that temporarily reduces its Darwinian fitness to the benefit of a social partner, is usually explained by kin selection |
| Dolivo & Taborsky 2015 (B) | Repeated donation game | Direct reciprocity, according to the decision rule ‘help someone who has helped you before’, reflects cooperation based on the principle of postponed benefits. |
| Donovan et al., 2020 | Prisoner's dilemma | When participants incur costs from social interaction (loss of resources or risk of harm), they must decide whether the costs are worth the potential benefits. In circumstances where participants interact repeatedly, mutual cooperation can offer long-term benefits to overcome short-term costs |
| Festucci et al., 2020 | Repeated donation game - Box ('open field') | The definition of prosocial behavior refers to actions aimed to provide help to individuals or groups, with or without expecting external rewards. |
| Fontes-Dutra et al., 2019 | Freeing task (tube) | Empathy is a complex phenomenon that could be understood in humans as the ability to understand and share the internal states of others, while generating an emotional response more appropriate to someone else’s situation than to one’s own. Therefore, empathy is frequently demonstrated through caring and helping behaviour toward others |
| Gerber et al., 2020 | Repeated donation game | "Here, we define cooperation in a descriptive, general sense as simultaneous or consecutive acting together of two or more individuals, without implying fitness costs and benefits to either partner. Reciprocity is defined as a helpful act apparently benefitting a receiver at immediate costs to the actor, which increases the probability to receive a helpful act in return |
| Hachiga et al,. 2018 | Freeing task (tube) | empathic action—that is, behavior in a rescuer intended solely to relieve distress in another. |
| Han et al., 2020 | Cooperation learning task | NA |
| Havlik et al., 2020 | Freeing task (tube) | NA |
| Hernandez-Lallement et al., 2015 | Prosocial choice task - Double T Maze | Pro-sociality,i.e.,the preference for outcomes that produce benefits for other individuals |
| Hernandez-Lallement et al., 2016 | Prosocial choice task - Double T Maze | NA |
| Hernandez-Lallement et al., 2020 | Not harming - Operant box | Empathy, the ability to share another individual’s emotional state and/or experience |
| Hosgorler et al., 2020 | Freeing task (soaked area) | NA |
| Kandis et al., 2018 | Freeing task (soaked area) | Empathy is the ability to recognize, process and respond to another’s emotional state |
| Karakilic et al., 2018 | Freeing task (soaked area) | "Empathy is defined as recognizing and internalizing the motivation of someone else's feelings, situation or behavior. Two fundamental types of empathy have been defined; emotional empathy and cognitive empathy. Emotional empathy (‘I feel what you feel’) is considered as a primitive behavior. Emotional contagion and imitation mimicry are considered within this context. Cognitive empathy (“I understand what you feel”) is considered as a higher-level process, involving cognitive processes such as glance acquisition and altruistic behaviors" |
| Kentrop et al., 2020 | Sharing task (lever presses) | Pro-social behavior, defined as behavior that is aimed to benefit others, is a key element in many aspects of everyday life. It is proposed to be driven by the motivation to maintain social relations and is hypothesized to emerge from different forms of empathy, from emotional contagion (i.e. the ability to experience and share emotions) to more cognitive forms of empathy like adopting the other’s point of view |
| Kozma et al., 2019 | Cooperation learning task (nose poke) | A crucial component of social cognition is the theory of mind, that is, the ability to make inferences on someone else's mental state (thoughts, emotions, or intentions) and predict his/her future behaviour based on social signals and the context of the situation. For example, even if children with ASD are capable to recognize another person's goal and help him/her in attaining it, notwithstanding they are incapable to cooperate, suggesting that controlling the behaviour of two individuals to reach a common goal is a far more complex task |
| Li & Wood, 2017 | Repeated donation game | NA |
| Lopuch & Popik, 2011 | Cooperation learning task (nose poke) | Cooperation can be defined as the voluntary joint action of two or more individuals that benefits the recipient(s) (Brosnan & de Waal, 2002; Hamilton, 1964). An act of altruism benefits the recipient at the cost to the actor (Hamilton, 1964), while mutual benefits follow a joint action resulting in the simultaneous benefit for all individuals involved (Dugatkin, 2002; Krebs & Davis, 1993). |
| Marquez et al., 2015 | Prosocial choice task - Double T Maze | Animals often are prosocial, displaying behaviors that result in a benefit to one another |
| Oberliessen et al., 2016 | Prosocial choice task - Double T Maze | Beyond maximizing one's own material gains, fairness plays an important role in human behaviour and economic decision making. The tendency to base decisions not solely on selfish motives but considering others' outcomes as well has often been studied with economic games. Disadvantageous inequity aversion (IA) is a behavioural response to an inequitable outcome distribution yielding a smaller reward to oneself than to a conspecific, given comparable efforts to obtain the reward. |
| Raz, 2013 | Cooperation learning task | NA |
| Rutte & Taborsky, 2007 | Lever presses - Box ('open field') | Cooperation among unrelated individuals may be achieved by reciprocal altruism in which two or more individuals help each other in turn. The decision to cooperate is based on expected future help, which may be judged from past interactions. |
| Rutte & Taborsky, 2008 | Repeated donation game | The logic of reciprocal altruism is that the decision to pay some cost for the benefit of another individual is based on expected future help, which may be judged from past interactions. Cooperation among non-kin has been attributed sometimes to reciprocal altruism: Two or more individuals exchange behaviour that benefits the respective partner. |
| Sato et al., 2015 | Freeing task (soaked area) | Helping behavior is a prosocial behavior whereby an individual helps another irrespective of disadvantages to him or herself. (definition from conclusion: Empathy is thought to be divided into two major subcomponents: cognitive empathy and affective (emotional)àç empathy (de Waal 2008; Hoffman 2000). Cognitive empathy is the ability to understand the thoughts, feelings, and desires of other individuals, and emotional empathy is the ability to share the emotional states of other individuals). Sensitivity to the emotions of conspecifics, through empathy or emotional contagion, is important to facilitate smooth communication with others and is necessary for an adaptive social life. As opposed to antisocial behavior, prosocial behavior is socially desirable behavior that benefits other individuals |
| Schmid et al., 2017 |  | In an iterated prisoner’s dilemma game, individuals base their decision to provide help to a partner or not on the latter’s previous help provided to them (direct reciprocity; Axelrod & Hamilton, 1981) or to others (indirect reciprocity; Nowak & Sigmund, 1998). Alternatively, an individual may help someone if it had previously received help from somebody else (generalised reciprocity |
| Schneeberger et al., 2012 | Repeated donation game | NA |
| Schonfeld et al., 2020 | Prosocial choice task - Double T Maze | The choice for a mutual reward does not lead to any direct benefit for the rat that makes the decision, it is assumed that a preference for mutual rewards reflects rodent social behavior, specifically prosocial decision making |
| Schwartz et al., 2017 | Freeing task (soaked area) | Empathic action—aiding a recipient despite cost to a donor— |
| Schweinfurth & Taborsky, 2016 | Repeated donation game | Three forms of reciprocal cooperation among animals have been described: direct reciprocity where individuals help those that have helped them before; generalized reciprocity, where the decision to help a social partner is based on help received from someone else; and indirect reciprocity where the decision to help a partner is dependent on the helpfulness of this partner towards others. |
| Schweinfurth & Taborsky, 2017 | Repeated donation game | Individuals showing direct reciprocity help those that have previously helped them (the concept of reciprocity is applied only to situations where the same social service or commodity is returned to the same social partner in a similar context) |
| Schweinfurth & Taborsky, 2018 (A) | Repeated donation game | Negotiation about mutual help might be an important mechanism responsible for the evolution of cooperation because it can generate greater fitness rewards and lead to higher levels of cooperation than kin selection. Cooperation among conspecifics, such as one individual helping another, is common in animals |
| Schweinfurth & Taborsky, 2018 (B) | Repeated donation game | The evolution and maintenance of cooperative interactions between unrelated individuals can be explained by the reciprocal trading of given and received help |
| Schweinfurth & Taborsky, 2018 (C) | Sharing task (stick pulling) | altruistic behaviours, entailing immediate costs without compensation by immediate benefits, are widespread in nature |
| Schweinfurth & al., 2019 | Repeated donation game | Evolutionary theory predicts individuals will behave in their own interest. Nevertheless, in many species individuals cooperate by providing costly help to others. The evolution of such cooperation depends on its costs and benefits. |
| Schweinfurth & Taborsky, 2020 | Prisoner's dilemma | The reciprocal exchange of help between social partners can lead to stable cooperation by taking turns |
| Silberberg et al., 2014 | Freeing task (soaked area) | empathically motivated behavior (altruism) consists of actions in one animal (the donor) to redress the perceived needs of another (the recipient). |
| Silva et al., 2020 | Freeing task (tube) | Empathy is the ability to (a) be affected by and share the emotional state of another; (b) assess the reasons for the other’s state; and (c) identify with the other, adopting their perspective. |
| Tomek et al., 2019 | Freeing task (tube) | NA |
| Tomek et al., 2020 | Freeing task (tube) | Here, we define prosocial behavior as those that occur with the intent to interact with others. One important aspect of prosocial behavior is empathy, the ability to perceive and understand the emotions or situations of others. |
| Tsoory et al., 2012 | Cooperation learning task | Cooperation is broadly defined as a situation in which an individual’s outcomes depend not only on its own behaviors but also on the behaviors of others |
| Ueno et al., 2019 (A) | Freeing task (tube) | "Prosocial behaviour comprises actions that benefit others and is said to include informing, comforting, sharing, and helping. Helping behaviour, a form of prosocial behaviour, involves acting for the benefit of others (e.g. rescuing others from difficult situations) in the absence of reward" |
| Ueno et al., 2019 (B) | Freeing task (tube) | Expressing socially desirable behaviours for conspecifics without external compensation is called prosocial behaviour |
| Viana et al., 2010 | Prisoner's dilemma in a double T-Maze | a cooperative act constitutes a truly altruistic behaviour emerging from a reward value attributed to the perception of benefit to others. Alternatively, from a strictly economic perspective, it is proposed that animals cooperate whenever it entails a benefit, either immediate or in the future, regardless of the consequence of its action to the other interacting individual |
| Wood et al., 2016 | Prisoner's dilemma in operant box | direct reciprocity is a dyadic interaction, representing the repeated reciprocal exchange of equivalent benefits between two parties. When delivering a benefit to their partner, each participant experiences a temporary net cost, which is exceeded by the benefit they subsequently receive from a partner working on their behalf |
| Yamagishi et al., 2020 (A) | Freeing task (soaked area) | prosocial behavior (e.g., helping, consolation, and food sharing), voluntary behavior through which they provide benefits to other individuals. Prosocial behavior is considered to be motivated by empathy, or the ability to share and understand others’ emotions |
| Yamagishi et al., 2020 (B) | Freeing task (soaked area) | Observing others’ emotional responses elicits emotional reactions from observers. Such an ability to share others’ emotions is called empathy |
| Yuksel et al., 2019 | Freeing task (soaked area) | Empathy is the recognition and internalization of someone else’s feelings, condition, or behavior. |

**Updated search – Characteristics of the included studies (2021-2023)**

**Table 7.** Results of all Included Studies – Animals (updated search)

| **Reference** | **Type of rodent** | **#rodents** | **#males** | **#females** | **Age** | **Strain** | **Weight(g)** | **Water restriction** | **Food restriction** |
| --- | --- | --- | --- | --- | --- | --- | --- | --- | --- |
| Asadi et al., 2021 | Rats | 30 | 30 | NA | Adult | Wistar | NA | No | No |
| Ben-Ami Bartal & al., 2021 | Rats | 83 | 83 | NA | Adult | Long-Evans + Sprague Dawley | NA | No | No |
| Breton et al., 2022 | Rats | 90 | 90 | NA | Ado: PND32 Adult: PND 60-90 | Long-Evans + Sprague Dawley | NA | No | No |
| Conde-Moro et al., 2022 | Rats | 38 | 38 | NA | 3 months | Lister Hooded | 250-300g | No | Yes |
| Cox et al., 2022 [A] | Rats | 18 | 18 | NA | NA | Sprague-Dawley | 250-275g | No | Yes |
| Cox et al., 2022 [B] | Rats | 16 | 8 | 8 | NA | Sprague-Dawley | 250-275g | No | Yes |
| de Carvalho et al., 2020 | Rats | 10 | 10 | NA | 3 months | Wistar | NA | Yes | No |
| Gachomba et al., 2022 | Rats | 86 | 74 | 12 | 3-3.5 months | Sprague-Dawley | 226-250g | No | No |
| Heslin et al., 2021 | Rats | 43 | 43 | NA | 9 months | Sprague-Dawley | NA | NA | NA |
| Joushi et al., 2022 | Rats | NA | NA | NA | PND34 | Wistar | NA | No | No |
| Kalamari et al., 2021 | Rats | NA | NA | NA | adults | Wistar | NA | No | No |
| Misiolek et al., 2023 | Mice | 89 | 45 | 44 | 10-12 weeks | C57BL/6 | 17-29 g | No | Yes |
| Paulsson & Taborsky, 2021 | Rats | 25 | NA | 25 | NA | Norway | NA | No | No |
| Scheggia et al., 2022 | Mice | 68 | NA | NA | 2-6 months | C57BL/6J | NA | No | Yes |
| Schweinfurth, 2021 | Rats | 52 | NA | 52 | Adult | Norway | 256-408g | No | No |
| Segura et al., 2019 | Rats | 8 | 8 | NA | Adult | Wistar | 228-264g | No | Yes |
| Sen et al., 2021 | Rats | 16 | 16 | NA | Adult | Sprague-Dawley | NA | No | No |
| Shima et al., 2022 | Mice | 16 | 16 | NA | 8 weeks | C57BL/6 | NA | No | No |
| Subhadeep et al., 2022 | Rats | 32 | 32 | NA | Adult | Wistar | 200-240g | No | No |
| Wan et al., 2021 | Rats | 6 | NA | 6 | NA | Sprague-Dawley | NA | No | Yes |
| Wu et al., 2023 | Rats | 24 | NA | 24 | 9-20weeks | Long-Evans | 200-300g | No | Yes |

**Table 8.** Results of all Included Studies – Housing Conditions and Apparatus (updated search).

| **Reference** | **Housing (per cage)** | **Relationship** | **Light-Dark Cycle** | **Enrichment** | **Operant Paradigm** | **Video/vocalization recording** | **Can animals see/hear/smell each other?** |
| --- | --- | --- | --- | --- | --- | --- | --- |
| Asadi et al., 2021 | paired | littermates | 12:12 dl lights on @ 7am | No | Freeing task (soaked area) | Videorecording | NA |
| Ben-Ami Bartal & al., 2021 | paired | NA | 12:12 dl | No | Freeing task (tube) | Videorecording | Yes |
| Breton et al., 2022 | paired | Littermates (ado) | 12:12 dl lights on @ 7am | No | Freeing task (tube) | Videorecording | Yes |
| Conde-Moro et al., 2022 | paired | NA | 12:12 dl | No | Cooperation | No | Yes |
| Cox et al., 2022 [A] | paired | NA | reversed 12:12dl lights on @ 18:00 | No | Freeing task (soaked area) | Ultrasonic voc | NA |
| Cox et al., 2022 [B] | paired | NA | Reversed 12:12dl | No | Freeing task (soaked area) | Ultrasonic voc | NA |
| de Carvalho et al., 2020 | paired | NA | 12:12 dl | No | Cooperation | Videorecording | Yes |
| Gachomba et al., 2022 | paired | NA | Reversed 12:12 dl lights off @ 8:30am | No | Prosocial choice task | Videorecording + Ultrasonic voc | Yes |
| Heslin et al., 2021 | paired | NA | Reversed 12:12 dl | No | Freeking task (tube) | No | Yes (smell and hear) |
| Joushi et al., 2022 | 6-9 per cage | littermates | 12:12 dl | Yes | Prosocial choice task | Videorecording | Yes |
| Kalamari et al., 2021 | 10 males per cage | littermates | Reversed 12:12dl lights on @ 20:00 | Yes | Freeing task (tube) | Ultrasonic voc | Yes |
| Misiolek et al., 2023 | paired | littermates | 12:12 dl lights on @ 7am | No | Prosocial choice task | Videorecording | Yes |
| Paulsson & Taborsky, 2021 | 5 sisters | littermates | Reversed 12:12 lights off @ 8am | No | Repeated donation game | Videorecording | Yes |
| Scheggia et al., 2022 | 2-4 per cage | littermates | 12:12 dl lights on @ 7am | No | Sharing task | Videorecording | Yes |
| Schweinfurth, 2021 | 5 per cage | NA | Reversed 12:12dl lights on @ 20:00 | Yes | Repeated donation game | No | Yes |
| Segura et al., 2019 | single | NA | 12:12 dl lights on @ 7am | No | Cooperation | Videorecording | Yes (except in opaque condition) |
| Sen et al., 2021 | paired | NA | 12:12 dl | No | Freeing task (soaked area) | NA | NA |
| Shima et al., 2022 | NA | NA | 12:12 dl lights on @ 8am | No | Freeing task (soaked area) | NA | NA |
| Subhadeep et al., 2022 | paired | NA | 12:12 dl lights on @ 6am | No | Freeing task (tube) | Ultrasonic voc + Videorecording | Yes |
| Wan et al., 2021 | paired | NA | 12:12 dl | No | Freeing task (tube) | NA | Yes |
| Wu et al., 2023 | single | NA | 12:12 dl lights off @ 18:00 | No | Freeing task (tube) |  | Yes |

**Table 9.** Results of all Included Studies – Methodology (updated search).

| **Reference** | **Duration (in min)** | **# of Testing Days** | **Habituation** | **Pretraining** | **Reward** | **Control Group** | **Other Behavioral Tests** | **Drugs or Surgical Intervention** |
| --- | --- | --- | --- | --- | --- | --- | --- | --- |
| Asadi et al., 2021 | 5 | 12 days | No | No | NA | No | NA | NA |
| Ben-Ami Bartal & al., 2021 | 40-60 | 12 days | Yes | No | social contact | Yes | Boldness, open field | NA |
| Breton et al., 2022 | 40-60 | 12 days | Yes | No | social contact | No | Open field | NA |
| Conde-Moro et al., 2022 | Until reached criterion | At least 2 consecutive days | Yes | Yes | pellet | Yes | Open field, elevated plus maze, water and food competition tests | Animals were chronically implanted with 2 sets of recording electrodes+ implantation of cannulas for micro injection |
| Cox et al., 2022 [A] | 5 | 2 sessions per day for 8 days | No | No | social contact | No | Social reward place conditioning | AAV8-CaMKIIα-enhanced green fluorescent protein or inhibitory DREADD virus, AAV8-CaMKIIα-hM4D(Gi)- mCherry |
| Cox et al., 2022 [B] | 5 | 2 sessions per day for 8 days | No | No | social contact | No | Social reward place conditioning | NA |
| de Carvalho et al., 2020 | 60 | 10 sessions | No | Yes | water | Yes | NA | NA |
| Gachomba et al., 2022 | 40 | 5 sessions | Yes | Yes | pellet | No | Food competition test | NA |
| Heslin et al., 2021 | 8 | 24 days (2 blocks of 12 sessions) | Yes | Yes | social contact | Yes | NA | NA |
| Joushi et al., 2022 | 6 forced+ 25 free choice trials | 12 sessions | Yes | Yes | 2 sucrose pellets | Yes | NA | Oxytocin |
| Kalamari et al., 2021 | 5-10 | NA | Yes | Yes | sucrose pellets or social contact | Yes | boldness test | NA |
| Misiolek et al., 2023 | Until reached criterion | 4 days | Yes | Yes | 2 chocolate chips | No | Social conditioned place preference, affective state discrimination | NA |
| Paulsson & Taborsky, 2021 | 7 | 3 sessions | No | Yes | oat flake | Yes | NA | NA |
| Scheggia et al., 2022 | 40-120 | Reached criterion for 3 consecutive days. | No | No | 14mg of test diet | Yes | Dominance tube test, observational fear conditioning | AAV5-CamKIIa-mCherry Virus injected into BLA. |
| Schweinfurth, 2021 | 7 | 18 sessions | No | Yes | 1 oat flake | No | NA | NA |
| Segura et al., 2019 | 4 blocks of 4 min | 30 sessions | No | Yes | 1 pellet | No | NA | NA |
| Sen et al., 2021 | 5 | 12 days | Yes | Yes | social contact | Yes | Open field, elevated plus maze, force swimming test | NA |
| Shima et al., 2022 | 3 | 1 day | No | Yes | NA | Yes | NA | NA |
| Subhadeep et al., 2022 | 30 | 8 days | Yes | Yes | social contact | Yes | Boldness test | Ibotenic acid (1 μg/μl/site)infused into the vSUB bilaterally |
| Wan et al., 2021 | 30 | 4-13 sessions (8.5 avg) | Yes | Yes | food or social contact | Yes | NA | NA |
| Wu et al., 2023 | 30 trials | NA | Yes | Yes | social contact | Yes | Observational distress test | Microelectrode implantation |

**Table 10.** Result of all Included Studies - Performance and Behavioral Analyses (updated search).

| **Reference** | **Performance index** | **Behavioral Analyses** |
| --- | --- | --- |
| Asadi et al., 2021 | # of door openings (# responses in HMC group in last 2 sessions was ↑ than LMC group. Rats that received higher maternal care early in life responded faster to rescue conspecific.) | NA |
| Ben-Ami Bartal & al., 2021 | # of door openings (rats released cagemates of the same strain, but not strangers of an unfamiliar strain, demonstrating an ingroup bias for prosocial behavior.) | Rats in HBT ingroup condition were more active in total. Rats in ingroup condition also spent more time in the area around the restrainer |
| Breton et al., 2022 | # of door openings (adults and ado tested with ingroup members were motivated to release cagemates. Unlike adults, ado released outgroup members as expressed by a sig. ↑ in the % of door-openings and ↓ latency. Nearly all ado (n = 6/8) consistently released trapped outgroup member vs 0/16 in adults.) | Ado in ingroup condition demonstrated movement patterns that reflect ↑ interest in the trapped rat, which may indicate motivation to release the trapped cagemate. Ado in both conditions were more active and spent more time near the trapped rat than adults. |
| Conde-Moro et al., 2022 | # of coordinated climb (all pairs learned to climb onto the platform to mutually obtain a reward and reached criterion between sessions 4-10. For each pair, the rat that climbed onto the platform sig. more times in first place (initiated more cooperation trials) was classified as the leader while the partner was classified as follower.) | Leader rats spent sig. more time in the open arms of the elevated plus-maze showing ↓ levels of anxiety. Follower rats showed a sig. ↑ level of social dominance than leader rats during these tests. |
| Cox et al., 2022 [A] | # of door openings (Inhibition of the AI sig. blunts release behavior during social contact-independent targeted helping task. The change in Observers’ helping behavior due to AI inhibition correlates to an ↑ in distress of the Target as measured by USV.) | Target rats of Observer partners that received B/M infusions had a sig. larger proportion of their total USV calls fall within the distress range compared to Targets on days where their corresponding Observers received PBS control. |
| Cox et al., 2022 [B] | # of door opening (Rats released a distressed conspecific at similar rates in the 3 timepoints evaluated) | Female Targets had a sig. larger proportion of their total USV calls fall within the distress range, and sig. fewer within the prosocial range compared to other groups. |
| de Carvalho et al., 2020 | # of simultaneous press (↓ in rates and proportion of coordination were observed at larger FR values. Successive ↑ in FR requirements produced systematic ↑ in post reinforcement pauses. Response requirement and reinforcement rates were critical determinants of coordinated responding maintained by FR schedules of mutual reinforcement.) | NA |
| Gachomba et al., 2022 | # of both-choice reward (Rats’ prosocial preferences in food-foraging contexts emerged over the testing sessions independently of familiarity or sex. Male rats displayed similar levels of prosociality when interacting with their cagemates or unfamiliar conspecifics.) | Both groups acquired a preference for prosocial option over the days, but social hierarchy drastically modulated the emergence of this choice. Dominant animals acquired faster prosocial tendencies and reached higher prosociality levels than submissive decisionmakers. Submissive recipients are more attentive: they display more direct gazing prior to choice and increase proximity to their focals, specifically when decision-makers are going to be selfish (i.e., following them around the choice area). Dominant decisionmakers might respond to these cues by showing ↑ social attention to their recipients which is reflected in ↑ sniffing time directed to the animal that needs help. All USVs recorded were of the 50-kHz family (no alarm calls were observed) |
| Heslin et al., 2021 | # of door openings (Choices made by the 19 subjects indicated an overall preference for selecting the nonrestrained rat chamber. Subjects initially preferred choosing locations that resulted in socialization opportunities, particularly with nonrestrained animals.) | NA |
| Joushi et al., 2022 | # of both-choice reward (Sig. ↑ proportion of BR choices in partner condition vs toy condition. % of BR choices in partner condition was sig. ↓ in MS group vs CTRL group. Being exposed to EE enhanced BR choices in partner condition in MS+EE group vs MS group. Exposure to EE reversed the impairment in mutual reward preferences caused by MS. % of BR choices in partner condition was sig.↓ in MS.saline group vs CTRL.saline group.) | NA |
| Kalamari et al., 2021 | # of door openings (MD rats seemed less motivated to liberate a trapped cagemate as seen by a lower number of completed ratios, but this effect was (just) not sig., although the effect size was quite large.) | Housing did not affect the emission of alarm calls before door opening. The emission of 22 kHz and appetitive calls were comparable between standard and complex housed rats. |
| Misiolek et al., 2023 | # of both-choice reward (In female mice, the preference for the prosocial compartment sig. ↑ while males appeared to show no change from their initial choices.) | NA |
| Paulsson & Taborsky, 2021 | # of stick pulling (Rats showed more reaching behaviours when a partner capable of providing food was present than when none was present but not in the presence of only a partner without food that it could have fetched for the focal subject.) | NA |
| Scheggia et al., 2022 | # of altruistic choices (Mice intentionally engaged in choices that favor another conspecific or only themselves. Prosocial actions, even if they required more effort or had no direct benefit to the actor mouse, were more generally observed toward familiar, hungry males with the highest hierarchical distance to the actor.) | Dominance test: most mice that displayed a preference for selfish over altruistic choices were subordinate to their recipient and belonged to an intermediate rank. |
| Schweinfurth, 2021 | # of cooperative choices (Rats distinguished between both conditions and provided more food to able food-providing partners vs unable partners that could no provide food as the device was blocked. Subjects helped able and willing partners more often than able and unwilling partners. Unable partners attempted to help by pulling the stick that was connected to the blocked platform, suggesting that they had cooperative intentions. Rats seem to not consider their partner's intention to help but base their decision on outcomes or abilities to help.) | NA |
| Segura et al., 2019 | #of coordinated actions (Rats from Pairs 1 and 2 coordinated their activities only when the reinforcement ratio was larger in the mutual option (4:1) while Pairs 3 and 4 failed to coordinate their actions.) | NA |
| Sen et al., 2021 | # of door openings (Mean opening door latency ↓ progressively in all animals. Door opening times after chronic restraint stress protocol were compared, no sig. difference found between the groups.) | No sig. difference between the groups in the open field test and elevated plus maze test. |
| Shima et al., 2022 | Light-intensity exercise enhances helping behavior with upregulated levels of BDNF mRNA in the insular cortex. | NA |
| Subhadeep et al., 2022 | # of door openings (On the 5th day, 4 out of 8 (50%) VSLfree rats could successfully open the restrainer door, which did not improve further in the subsequent days. On the 8th day, only 2 out of 8 (25%) VSLfree rats successfully opened the door.) | During the task, both 22-kHz and 50-kHz USV calls were emitted by the pair of rats in the arena. Most of the 22-kHz calls were emitted before the restrainer door was opened. 50-kHz calls were emitted after the free rats successfully opened the door. The # of 22-kHz calls emitted by the VSL rats before door-opening on day 1 was the least, which gradually increased in the subsequent sessions. VSL rats continued to emit more 22-kHz calls and fewer 50-kHz calls even after door-opening. |
| Wan et al., 2021 | # of door opening containing the congener vs the food (Rats chose food and social release with similar latencies and rats willingly share food with their social partner, even if it comes at a cost to the individual.) | NA |
| Wu et al., 2023 | # of door opening (8/12 rats opened the restrainer more often when a conspecific was in the restrainer.) | NA |

**Table 11.** Results of all Included Studies - Other Analyses (updated search)

| **Reference** | **Sex Differences** | **Age Differences** |
| --- | --- | --- |
| Breton et al., 2022 | NA | Adults released trapped ingroup members, but ado rats helped both ingroup and outgroup members, suggesting ingroup bias emerges in adulthood. |
| Cox et al., 2022 [B] | No sex differences | NA |
| Misiolek et al., 2023 | Females, but not males, C57BL/6 mice showed significant preference for prosocial behavior toward a familiar partner. | NA |
| Scheggia et al., 2022 | Males are more prosocial than females. | NA |

*Articles that had no reported information regarding results in this table were excluded from it*

**Table 12.** Results of all Included Studies – Reported definition (updated search)

| **Reference** | **Task** | **Reported definition** |
| --- | --- | --- |
| Asadi et al., 2021 | Freeing task (soaked area) | Empathy has been defined as the ability to vicariously experience a shared affective state as another person, coupled with provoked caring and concern for others’ good. |
| Ben-Ami Bartal & al., 2021 | Freeing task (tube) | acting with the intention of benefiting others or improving their well-being + helping others in need |
| Breton et al., 2022 | Freeing task (tube) | Prosocial actions are any that occur with the intention of benefiting others or improving their well-being |
| Conde-Moro et al., 2022 | Cooperation | cooperation: a powerful way of improving the access to resources and require a precise synchronization of animal activities |
| Cox et al., 2022 [A] | Freeing task (soaked area) | Empathy is the capacity to share the feelings of another and generate an appropriate response to those shared feelings |
| Cox et al., 2022 [B] | Freeing task (soaked area) | Empathy is a complex suite of behaviors that works to convey an understanding of the affective states of others |
| de Carvalho et al., 2020 | Cooperation | NA |
| Gachomba et al., 2022 | Prosocial choice task | performing actions that benefit others |
| Heslin et al., 2021 | Freeking task (tube) | Prosocial behavior is any behavior that provides a benefit to another individual, with little or no cost to the actor |
| Joushi et al., 2022 | Prosocial choice task | Pro-sociality, i.e., the preference for outcomes that produce benefits for other individuals |
| Kalamari et al., 2021 | Freeing task (tube) | behavior that benefits others, |
| Misiolek et al., 2023 | Prosocial choice task | Prosocial behavior, defined as acting to meet the perceived need of another individual, is regarded as the highest form of empathy |
| Paulsson & Taborsky, 2021 | Repeated donation game | Reciprocal altruism or “reciprocity,” where a cost is accepted by an individual to provide a service to a social partner for a delayed benefit, |
| Scheggia et al., 2022 | Sharing task | NA |
| Schweinfurth, 2021 | Repeated donation game | Reciprocity is the selective helping of those who were cooperative before |
| Segura et al., 2019 | Cooperation | Cooperative behavior has been defined as “joint action for mutual benefit” |
| Sen et al., 2021 | Freeing task (soaked area) | Empathy, can be defined as understanding and internalizing someone else’s emotions, current situation or behaviour |
| Shima et al., 2022 | Freeing task (soaked area) | NA |
| Subhadeep et al., 2022 | Freeing task (tube) | empathy: the ability to understand and share the emotions of others. |
| Wan et al., 2021 | Freeing task (tube) | Pro-social behavior has been defined as behavior that produces benefits for another, sometimes even at a cost to the individual |
| Wu et al., 2023 | Freeing task (tube) | Emotional contagion, the ability to experience the distress of others, is closely associated with prosocial behavior that benefits others |
